# *Staphylococcus aureus* wall teichoic acid structure dictates virulence potential and resistance to host defenses

**DOI:** 10.1126/sciadv.aef7070

**Published:** 2026-10-21

**Authors:** Timothy J. Enroth, Eunjeong Lee, Elan Z. Eisenmesser, Alexander R. Horswill

**Affiliations:** ^1^ Department of Immunology and Microbiology, University of Colorado School of Medicine, Aurora, CO 80045, USA.; ^2^ Department of Biochemistry and Molecular Genetics, University of Colorado School of Medicine, Aurora, CO 80045, USA.; ^3^ Ochang Bio and Environmental Research Institute, KBSI, 162 Yeongudanji-ro, Ochang-eup, Cheongwon-gu, Cheongju-si 28119, Chungcheongbuk-do, Korea.; ^4^ Department of Veterans Affairs Eastern, Colorado Healthcare System, Aurora, CO 80045, USA.

## Abstract

*Staphylococcus aureus* is a formidable pathogen and the leading cause of skin and soft tissue infections. We recently showed that the highly conserved *sasF* gene, encoding for an uncharacterized sortase-anchored surface protein, is up-regulated when *S. aureus* colonizes skin. Here, we demonstrate that SasF is an enzyme that has glycoside epimerase activity, which is required for *S. aureus* resistance to skin fatty acids. Mutants of *sasF* are highly impaired in skin colonization and acute infection models. The SasF enzyme is crucial for maintaining the structure of wall teichoic acids and proper *S. aureus* surface chemistry. To our knowledge, this is the first report of a bacterial surface epimerase in this pathogen, suggesting that SasF could be a therapeutic target.

## INTRODUCTION

*Staphylococcus aureus* is a highly successful bacterial pathogen that can cause a diverse array of infections. It is the dominant cause of skin and soft tissue infections at 76% of reported cases in the US (*1*). *S. aureus* virulence factors combined with its capacity to colonize up to a third of healthy adults (*2*) make this pathogen uniquely capable of infecting not only the skin but also, practically, every site of the body. Antibiotic resistance in *S. aureus*, particularly in the methicillin-resistant *S. aureus* (MRSA) strains, is widespread and complicates the treatment options. Endogenous infections arise from the same *S. aureus* strains that colonize the anterior nares (*3*, *4*), and it has been proposed that transient skin colonization from the nares is an important precursor to infection (*5*). Identifying mechanisms of how *S. aureus* survives on the skin is important for understanding the transition to subsequent infections (*6*).

In previous work from our lab, we assessed *S. aureus* genes important for skin colonization using RNA sequencing (RNA-seq) on healthy murine skin (*7*). One understudied gene, called *sasF*, drew our attention because it was highly up-regulated during colonization at both 5 and 24 hours postinoculation. The *sasF* gene encodes for an uncharacterized sortase-anchored surface protein that is nearly 100% conserved in *S. aureus* and is known to be anchored to the peptidoglycan layer (*8*–*10*), but the function of SasF has remained generally uncharacterized. In previous reports, *sasF* was found to be up-regulated on skin explants (*11*) and SasF protein was detected in the proteome during orthopedic implant infections (*12*). *sasF* has also been shown to be up-regulated in the presence of skin-relevant fatty acids, and a *sasF* mutant was found to be more susceptible to the antimicrobial effects of the same fatty acid (*13*). However, the specific function of the SasF protein and how it contributes to *S. aureus* physiology and pathogenesis remain unclear.

Here, we report an enzymatic role for SasF in actively changing the bacterial cell surface structures known as wall teichoic acids (WTAs). Work herein was conducted in a *S. aureus* LAC strain background, which is a member of the USA300 MRSA (ST8) lineage and has WTA molecules composed of polyribitol phosphate (*14*, *15*). WTA on the cell surface is highly glycosylated (*14*, *15*) and contains covalently bound d-alanine residues that impart a net positive cell surface charge, which contributes to resistance against antibiotics daptomycin and vancomycin as well as antimicrobial peptides (*16**–**18*). We show that SasF can alter the WTA structure, aiding *S. aureus* in resistance to skin fatty acids. WTA molecules have a reported role in fatty acid resistance (*19*), and this work provides new information on the mechanisms of this resistance. Additionally, we demonstrate that SasF is important in skin colonization and skin infection. Last, this work provides a deeper understanding of how WTA glycosides, in particular, might influence bacterial resistance to host innate stressors during colonization and infection.

## RESULTS

### SasF is important in skin colonization and fatty acid resistance

Previous work from our lab has shown that *sasF* is up-regulated in a mouse skin colonization model, fold changes of 2.1 and 3.5 at 5 and 24 hours postcolonization, respectively (Fig. 1A) (*7*). Considering this observation and other reports on SasF properties (*8*, *13*), we postulated that SasF is important for *S. aureus* skin colonization mechanisms and, potentially, the transition to infection. To test this question, we compared a *sasF* mutant constitutively expressing *lux^+^* with wild type (WT) in mouse skin colonization using in vivo bioluminescent imaging (IVIS). The *sasF* mutant has an early colonization defect (Fig. 1A), confirming our previous RNA-seq data (*7*). Notably, *sasF* transcription during skin colonization is correlated to the expression of the sortase-anchored skin corneocyte-binding adhesin SasG, whereas *sasF* expression is inversely correlated with the expression of certain WTA biosynthetic genes (Fig. 1A) (*7*).

**Fig. 1.**
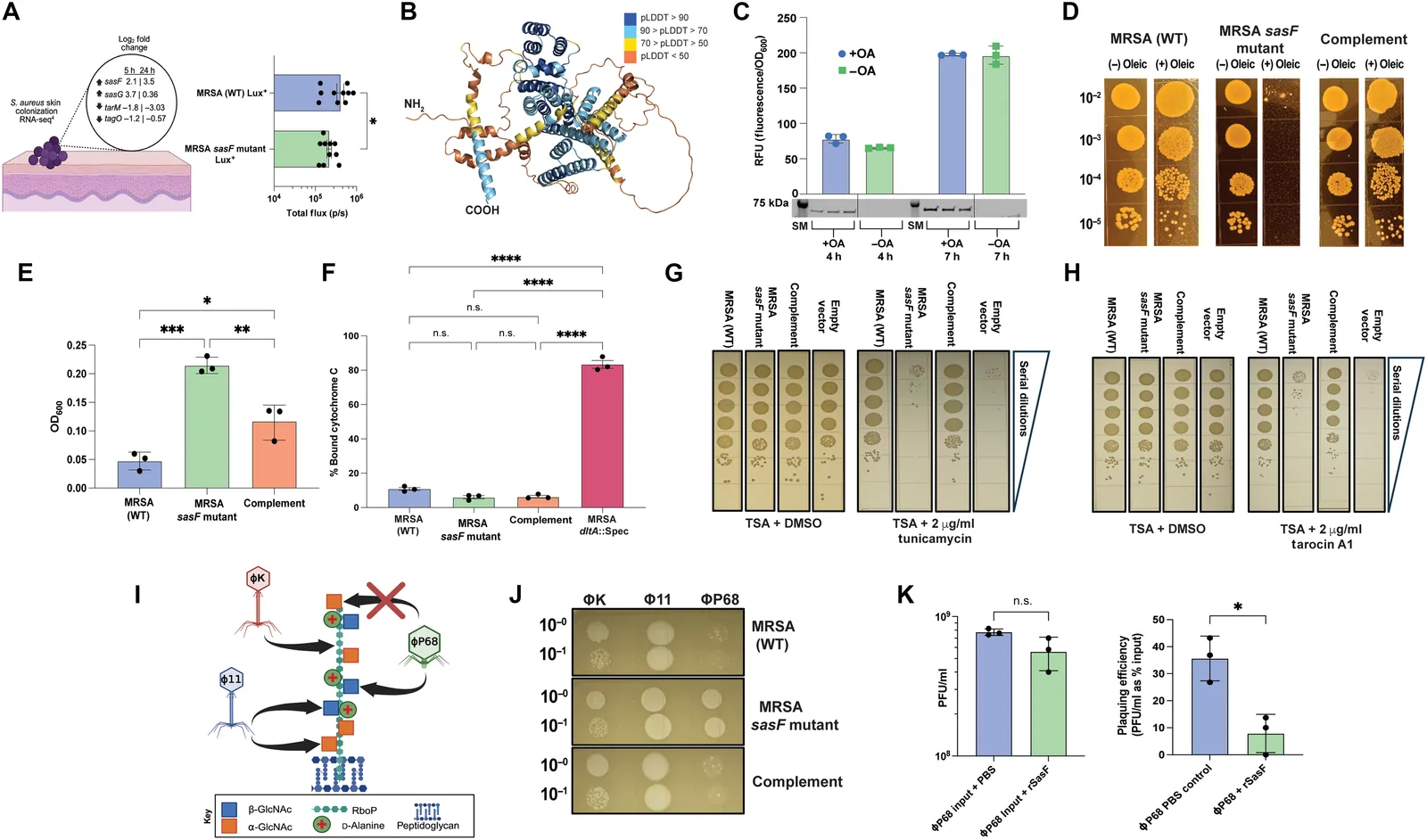
SasF modulates fatty acid resistance and general cell surface properties. (**A**) *sasF* is highly up-regulated during healthy skin colonization and is important for early (1-day) colonization. h, hours. (**B**) AlphaFold 3–predicted model (color coded by pLDDT confidence) of the SasF protein structure reveals an unconventional structure. (**C**) SasF production is posttranscriptionally regulated upon oleic acid challenge as oleic acid does not increase promoter activity despite markedly increasing SasF protein production. h, hours. (**D**) The MRSA *sasF* mutant is highly susceptible to oleic acid challenge compared with MRSA [wild type (WT)], and this phenotype is complementable. (**E**) The MRSA *sasF* mutant has a more hydrophilic cell surface as determined by *n*-hexadecane cell partitioning. (**F**) Cytochrome C binding to the bacterial cell of the MRSA *sasF* mutant is not different than MRSA (WT), suggesting that the differences in cell surface properties in the MRSA *sasF* mutant are not due to charge differences. (**G**) The MRSA *sasF* mutant is more susceptible to the WTA inhibitors tunicamycin and tarocin A1 (**H**) compared with MRSA (WT). (**I**) Bacteriophages can be used to partially assess WTA glycosylation patterns. (**J**) The MRSA *sasF* mutant displays altered phage binding as the MRSA *sasF* mutant is highly susceptible to ɸP68 infection. (**K**) Purified rSasF is sufficient to restore MRSA *sasF* mutant phage resistance against ɸP68. Shown are representative photos from three biological replicates. Statistics are measured by Student’s *t* test in (A) and (I) and an ordinary one-way analysis of variance (ANOVA) for (D). (A) Created in BioRender. T. Enroth (2026), https://BioRender.com/a9kw8yf. (I) Created in BioRender. T. Enroth (2026), https://BioRender.com/n59pnt4.

### SasF is not a surface adhesin

SasF is a highly conserved 71-kDa sortase-anchored protein of unknown function. Previous literature has referred to the protein as an adhesin (*20**–**23*). However, to our knowledge, there has been no direct experimental assessment of SasF in binding to extracellular matrix proteins or tissues. Most known adhesins in *S. aureus* have a relatively long protein structure that extends away from the cell surface (*24*). Structural modeling of SasF using AlphaFold 3 (*25*) suggests that the protein has a globular and relatively compact structure unlike that of typical *S. aureus* adhesins (Fig. 1B). We tested whether SasF might play a role in host matrix adhesion and observed that the *sasF* mutant can bind fibronectin slightly more than WT MRSA (fig. S1A) and that the *sasF* mutant does not bind fibrinogen differently than WT (fig. S1B). We also found no defect in adherence of the *sasF* mutant to differentiated immortalized N/TERT-2G human keratinocytes compared with WT (fig. S1C) (*26*). Additionally, we observed no difference in biofilm development for the *sasF* mutant (fig. S1D). Thus, in the models that we tested herein, SasF does not appear to contribute to host matrix binding, keratinocyte adherence, or biofilm formation as has been previously suggested (*20**–**23*).

### SasF modulates oleic acid resistance and cell surface properties

It has been reported that SasF may play a role in bacterial resistance to fatty acids (*13*), which are found in high concentrations within the stratum corneum of the skin (*27*). Although transcriptional studies have shown *sasF* to be up-regulated in the presence of fatty acids (*13*), more detailed regulatory analyses have not been performed. We assessed *sasF* promoter activity and production of SasF by Western in response to oleic acid, which can be up to 15% of the dry weight of the stratum corneum (*28*). For these analyses, we generated anti-SasF polyclonal antibodies raised against recombinant SasF, and we confirmed that these antibodies bind SasF from MRSA cell lysates (fig. S2A). Upon treatment of WT MRSA cells with 500 μM oleic acid, we did not observe a change in *sasF* promoter activity using a green fluorescent protein (GFP) reporter compared with untreated cells (Fig. 1C and fig. S2B). However, SasF protein production is markedly increased in the cells treated with oleic acid (Fig. 1C and fig. S2C), suggesting an unclear posttranscriptional regulatory mechanism. Therefore, MRSA responds to skin oleic acid challenge by up-regulating SasF expression posttranscriptionally.

We hypothesized that SasF in MRSA may be a crucial defense against oleic acid. When serial dilutions of the *sasF* mutant are spotted on medium containing oleic acid, we observed a substantial growth defect for the *sasF* mutant compared with WT, and this phenotype is complementable (Fig. 1D). Considering that fatty acids are highly hydrophobic in nature, we asked whether the general cell surface of the *sasF* mutant is changed in its chemistry. Using *n*-hexadecane partitioning to measure the hydrophilicity of the cell surface (*13*), we observed that the cell surface of the *sasF* mutant is significantly more hydrophilic than WT, which, again, could be complemented (Fig. 1E). As a control, we ruled out any interaction between purified rSasF and oleic acid using isothermal titration calorimetry where we observed no binding between the two (fig. S3). In all, our findings support a direct role for SasF in fatty acid resistance and modulation of the cell surface hydrophobicity.

### SasF is required for WTA-inhibitor resistance

To understand the SasF resistance mechanism, we next investigated WTAs, which are highly abundant extracellular polymers covalently linked to the extracellular peptidoglycan layer (*29*, *30*). Our reasoning is that WTA has been implicated in resistance to skin fatty acids and that they contribute to the general cell surface properties of the *S. aureus* cell (*17*, *19*, *31*), leading us to hypothesize that SasF may be involved in WTA homeostasis. We assessed the *sasF* mutant susceptibility and binding to Congo red, a negatively charged dye known to be excluded by WTA and whose binding is modulated by differences in cell surface charge (*32*). We observed an increase in Congo red resistance in the *sasF* mutant compared with MRSA (WT) (fig. S4). Notably, we did not observe differences in the binding of positively charged cytochrome C (*33*) to the *sasF* mutant compared with WT or the complement strain (Fig. 1F), ruling out any charge dependent effects of SasF.

Various chemical WTA biosynthesis inhibitors have been used previously to study aspects of WTA such as their structure, abundance, and glycosylation state. WTA biosynthesis can be significantly inhibited by the drug tunicamycin (*32*, *34**–**36*), and we speculated that, if there was a WTA defect for the *sasF* mutant, then we would observe increased susceptibility. In agreement with this hypothesis, we found the *sasF* mutant to be highly susceptible to tunicamycin (Fig. 1G and fig. S5) and that this phenotype is complementable. Tunicamycin has been recently shown to affect the transcription of numerous *S. aureus* genes, many of which are seemingly unrelated to WTA biosynthesis (*37*). Because of this, we aimed to rule out any pleotropic effects of tunicamycin by using a structurally different molecule that also inhibits WTA biosynthesis, called tarocin A1 (*38*). In agreement with the tunicamycin data, we observed the *sasF* mutant to also be highly susceptible to tarocin A1 (Fig. 1H). Together, our data are consistent with SasF being involved in MRSA WTA homeostasis.

### The *sasF* mutant WTA contains modified glycan decorations

*S. aureus* WTA molecules are complex polymers decorated with d-alanine residues and *N*-acetylglucosamine (GlcNAc) moieties, in either an α-1,4- or β-1,4- orientation (*14*, *15*, *39*). We tested whether any of these modifications might be altered in the *sasF* mutant, contributing to our tunicamycin and tarocin A1 observations. MRSA strains deficient in adding the d-alanine residues (*dlt* mutants) to teichoic acids become highly susceptible to cationic antimicrobial peptides, a phenotype that is recapitulated in other bacteria (*17*, *40*). Given that we did not observe any differences in cell surface charge by cytochrome C binding (Fig. 1F), we assessed whether the *sasF* mutant was more susceptible to the mouse cathelicidin-related antimicrobial peptide (mCRAMP) (*41*). We found that the *sasF* mutant was modestly more susceptible than WT (fig. S6).

There are a variety of phages against *S. aureus* that are known to use either the polyribitol phosphate backbone or the glycosides found decorating the WTA molecules as receptors (Fig. 1I) (*42*), providing a means to assess WTA structural differences. We determined whether SasF is involved in WTA glycosyl structural modification using bacteriophage plaque assays. We used three phages: ɸK, which uses the ribitol phosphate backbone of WTA as its receptor and serves as a control for teichoic acid abundance; ɸ11, which uses both α1,4- and β-1,4-GlcNAc; and ɸP68, which uses β-1,4-GlcNAc exclusively and whose infection is blocked by α1,4-GlcNAc. Using a plaque assay, we observed no difference in ɸK plaquing between WT and the *sasF* mutant, suggesting that the overall abundance of teichoic acids is not different between the strains (Fig. 1J). We observed no difference in plaque numbers for ɸ11 spotted onto the *sasF* mutant, suggesting that total carbohydrate abundance is not different for the *sasF* mutant compared with WT (Fig. 1J). However, we observed a significant increase in plaquing of ɸP68 on the *sasF* mutant compared with WT (Fig. 1J).

Next, we tested whether the SasF protein itself is sufficient to restore the *sasF* mutant’s resistance to ɸP68 bacteriophage. Purified SasF protein (rSasF) was used in a plaquing efficiency assay. In this assay, *S. aureus* the *sasF* mutant was treated with either rSasF or a buffer-only control, and plaquing by ɸP68 was assessed pre- and postaddition of rSasF. We observed that rSasF significantly restores resistance to ɸP68 compared with the control, suggesting that rSasF itself is sufficient for restoration of the *sasF* mutant resistance against ɸP68 (Fig. 1K). Together, these data suggest that there are not differences in cell surface charge, nor are there differences in the absolute levels of WTA decorating the *sasF* mutant cell. Rather, there may be changes in the composition or arrangement of glycosides decorating *sasF* mutant WTA molecules, and we aimed at pursuing this possibility further.

### SasF changes WTA glycoside chemical shifts

The susceptibility of MRSA *sasF* mutant to WTA inhibitors (tunicamycin and tarocin A1) and the altered bacteriophage plaquing suggest that there may be differences in WTA glycosylation. We used ^1^H-^13^C heteronuclear single quantum coherence (HSQC) two-dimensional (2D) nuclear magnetic resonance (NMR) to survey the chemistry of the WTA carbohydrates (Fig. 2A). Initially, we extracted ^13^C-enriched WTA from *sasF*, *tarM*, and *tarS* mutants. The *tarM* and *tarS* mutant strains are controls for defects in the addition of α1,4- and β-1,4-GlcNAc to the polyribitol phosphate WTA backbone, respectively (*14*, *15*). Given that rSasF can restore ɸP68 resistance upon direct addition, we reasoned that an NMR spectrum of the *sasF* mutant WTA pre- and postaddition of rSasF might provide insights on the glycosidic changes catalyzed by rSasF. In support of this, we observed multiple chemical shifts in the region where C3 and C4 are known to be located for glycoside ring resonances disappear upon rSasF addition (Fig. 2B and fig. S7). Using 2D-NMR spectra from *tarM* (which fails to add α1,4-GlcNAc to WTA) (Fig. 2C) and *tarS* mutants (which fails to add β-1,4-GlcNA to WTA) (Fig. 2D) as controls, we were able to tentatively assign the C1 anomeric carbon chemical shifts for each strain’s anomer of WTA-bound GlcNAc. We also ruled out whether rSasF is an anomerase as treatment results in no changes to the C1 chemical shifts (Fig. 2E).

**Fig. 2.**
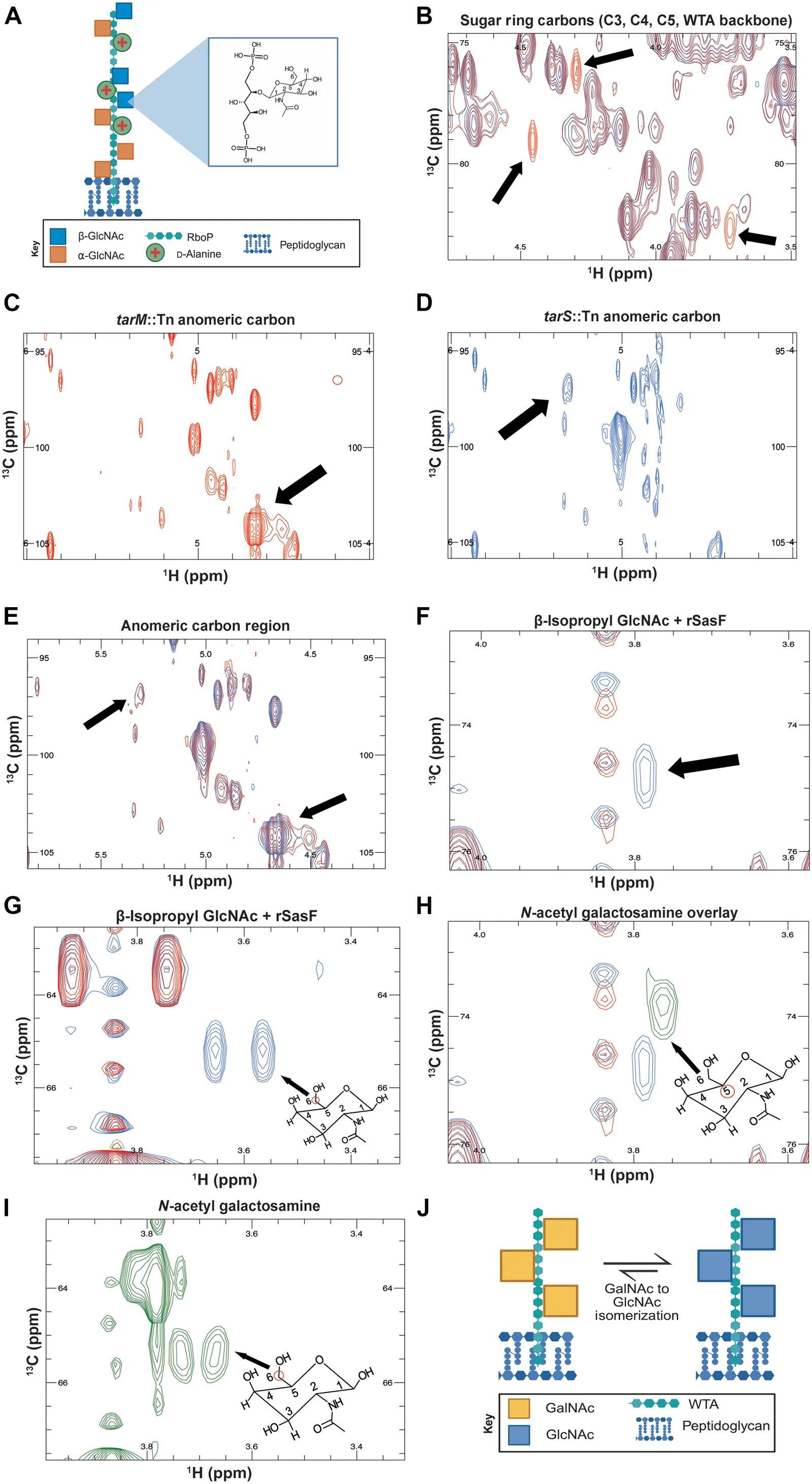
2D-NMR analyses of WTA from the MRSA *sasF* mutant reveal an altered structure and enzymatic activity of rSasF. (**A**) Model of *S. aureus* WTA showing glycoside chemical shifts focused on in the current figure. (**B**) HSQC spectra of WTA from the *sasF* mutant were obtained preaddition (red) and postaddition (blue) of 10 μM unlabeled rSasF overnight at room temperature, which shown distinct changes in chemical shifts corresponding to the GlcNAc carbon ring. (**C**) WTA extracted from *tarM::*Tn (which lacks α-1,4-GlcNAc) and *tarS::*Tn (which lacks β-1,4-GlcNAc) (**D**) are used to assign the anomeric carbon chemical shifts for WTA-bond GlcNAc. (**E**) The anomeric carbon chemical shifts for WTA preaddition (red) and postaddition (blue) of rSasF show no changes in position or intensity, suggesting that rSasF does not have glycosidase activity against WTA glycosides. (**F**) HSQC spectra of β-isopropyl GlcNAc WTA mimics were obtained preaddition (red) and postaddition (blue) of rSasF and incubation overnight at 37°C, which revealed a distinct chemical shift consistent with that of GalNAc C5 and suggest that rSasF exists in enzymatic equilibrium between GlcNAc and GalNAc. (**G**) Addition of rSasF (blue) to β-isopropyl GlcNAc (before addition of rSasF in red) results in a pair of protons consistent with those of GalNAc C6. (**H** and **I**) HSQC control spectra of pure GalNAc reveals that the observed chemical shifts for C5 (F) and C6 (G) in the isopropyl-GlcNAc samples treated with rSasF are consistent with C5 and C6 of GalNAc. (**J**) Proposed model for rSasF enzymatic isomerization of surface glycosides to GlcNAc. (A) Created in BioRender. T. Enroth (2026), https://BioRender.com/4irpjak. (J) Created in BioRender. T. Enroth (2026), https://BioRender.com/t4jg9je.

When analyzing the entire structure for the *sasF* mutant WTA pre- and postaddition of recombinant protein, we did not observe rSasF-mediated cleavage of glycosides from WTA as glycosidase activity would have been evident by the changes to GlcNAc C1 chemical shifts (Fig. 2E). Rather, our results suggest that rSasF may be epimerizing a WTA glycosde given the changes that were observed at the C3/C4 region upon rSasF addition to the *sasF* mutant WTA (Fig. 2B). This is supported by the observation that rSasF addition does not result in changes to known anomeric carbon chemical shifts (Fig. 2E). Upon addition of rSasF to WTA isolated from a *sasF* mutant, we observed the disappearances of chemical shifts at approximately δH = 4.45 parts per million (ppm) and δC = 79 ppm, δH = 4.3 ppm and δC = 76 ppm, and δH = 3.75 ppm and δC = 83 ppm. We also observed the disappearance of a chemical shift at approximately δH = 3.3 ppm and δC = 55 ppm (fig. S7). The observed chemical shifts in the *sasF* mutant are consistent with those of GalNAc, which led us to hypothesize that SasF is epimerizing GalNAc to GlcNAc on WTA. However, these chemical shift assignments are tentative, and we cannot definitively confirm that GalNAc is being epimerized to GlcNAc on WTA molecules without further experimentation.

### Small-molecule WTA mimics corroborate NMR findings

To determine whether SasF comprises epimerization activity, we repeated our initial WTA NMR experiment using small-molecule WTA mimics that have simpler HSQC spectra. Isopropyl glycoside has an O-linked isopropyl group covalently bound to C1 of either the α- or β-anomers of GlcNAc (figs. S8 and S9, respectively), similar to *S. aureus* WTA. We hypothesized that isopropyl-GlcNAc conversion to isopropyl-GalNAc might be observed upon rSasF addition, allowing assignment of product chemical shifts to GalNAc. In agreement with these hypotheses, upon rSasF addition to β-isopropyl-GlcNAc, we observed a chemical shift arise at approximately δH = 3.78 ppm and δC = 75 ppm (Fig. 2F), which is consistent with the chemical shift for GalNAc C5. We also observed two proton resonances at approximately δH = 3.58 ppm and δC = 65 ppm (Fig. 2G), which are consistent with the isopropyl-GalNAc diastereotopic protons at C6. We obtained an HSQC spectrum for pure GalNAc (nonisopropyl), and we observed a GalNAc chemical shift at nearly the same location as the chemical shift observed at δH = 3.78 ppm and δC = 75 ppm upon rSasF addition to isopropyl-GlcNAc, which likely corresponds to C5 (Fig. 2H). Similarly, we observed the same C6 proton chemical shifts in the GalNAc control spectrum at approximately δH = 3.7 ppm and δC = 65.5 ppm (Fig. 2I). Thus, our data together suggest that the equilibrium product of the GalNAc isopropyl glycosides with rSasF are likely isopropyl-GalNAc, given that multiple chemical shifts appear upon rSasF addition are consistent with those of pure GalNAc. Although, further structural studies would be needed to make a definitive assignment of the glycoside chemical changes upon rSasF treatment.

As a control, we compared our rSasF-treated isopropyl glycoside spectra with that of isopropanol to rule out glycosidic cleavage of GlcNAc from the isopropyl glycoside, which would result in free isopropanol in solution. This comparison showed no overlap between the new chemical shift observed at δH = 3.6 ppm and δC = 65 ppm and the chemical shifts from the isopropanol methine group, further confirming that rSasF does not cleave the glycoside at C1 to result in free isopropanol (fig. S10). This also suggests that the potential rSasF-mediated conversion of GalNAc to GlcNAc is likely occurring via epimerization. In agreement with this, we did not observe any changes to the anomeric carbon chemical shifts of the isopropyl glycosides treated with rSasF, further suggesting that SasF is not cleaving isopropanol from C1 or otherwise modifying the anomeric carbon. Therefore, when considering both the WTA NMR data and the isopropyl glycoside data, our results suggest that SasF can potentially epimerize WTA GalNAc to GlcNAc without glycosidic cleavage at C1 (Fig. 2J). This epimerization of GalNAc to GlcNAc may be occurring at C4 as GalNAc and GlcNAc only differ in proton orientation at C4. However, the exact glycoside substrate specificity of SasF would still need to be verified by additional methods.

### SasF has characteristics of known epimerases

We hypothesized that SasF would have a similar catalytic mechanism as other known GlcNAc 4-epimerases (*43**–**45*). There are reported GlcNAc 4-epimerases involved in metabolic processes that use nicotinamide adenine dinucleotide (oxidized form) (NAD^+^) as a cofactor (*45*, *46*) for cyclic reduction and oxidation. Many NAD-binding proteins contain a conserved Rossman fold domain (*47*), but there are no SasF conserved protein domains or folds that can be detected by domain and fold prediction software such as InterPro (*48*) and Pfam (*49*). However, there are reports of proteins without a Rossman fold that still bind NAD (*50**–**52*). We investigated whether SasF also uses NAD^+^/reduced form of NAD^+^ (NADH) starting with denaturing size exclusion chromatography to separate cofactors from the protein. We observed a characteristic NADH 340-nm absorbance peak in the flow-through that appears at the same elution volume as a NADH standard (Fig. 3A), supporting the hypothesis that rSasF may bind NADH similarly as known epimerases.

**Fig. 3.**
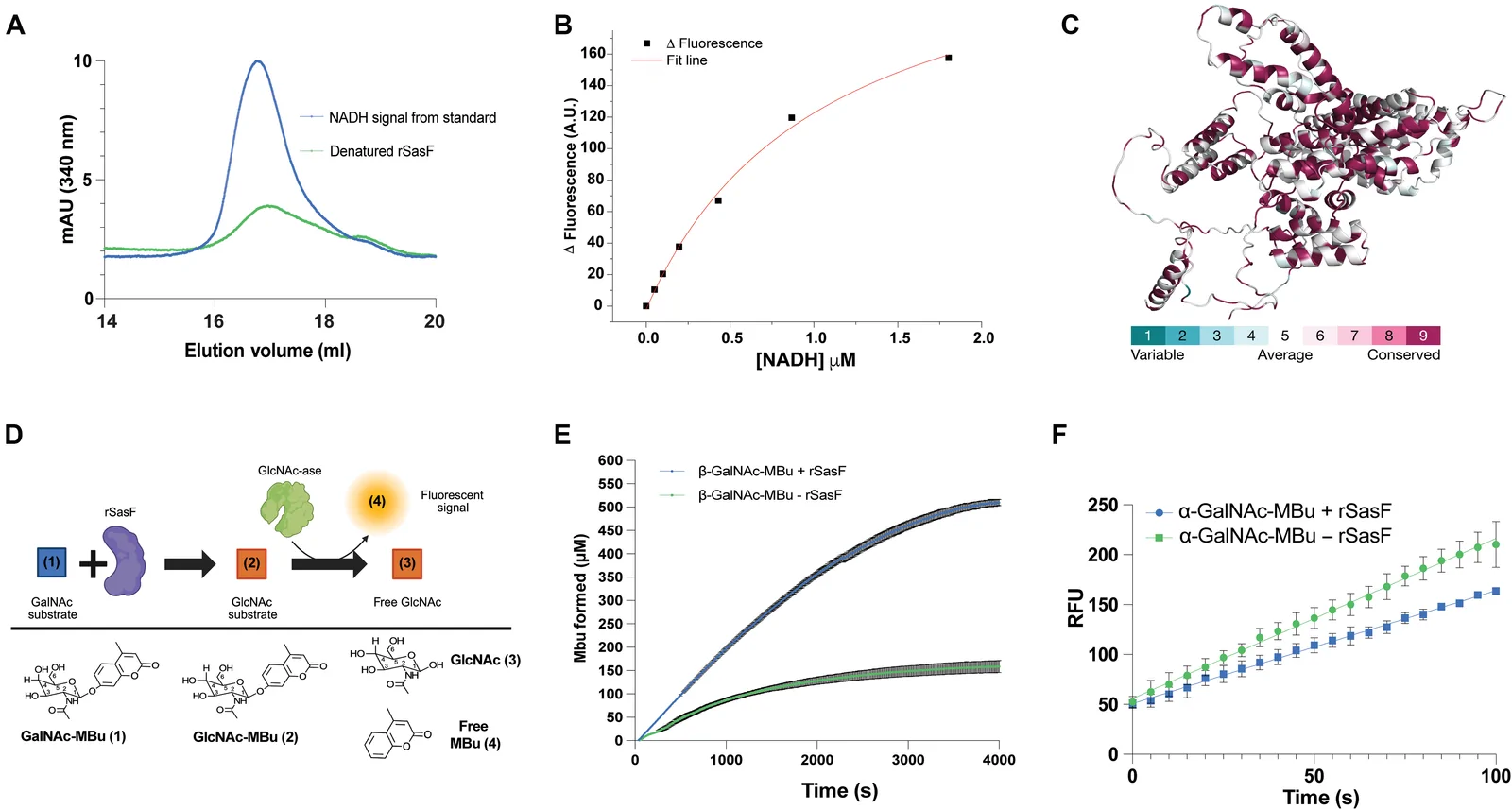
rSasF has characteristics of known isomerases and can isomerase GalNAc in vitro. (**A**) Chromatogram obtained following size exclusion chromatography of rSasF denatured with 8 M urea reveals a free NAD peak that matches that of a pure NAD standard, suggesting that rSasF uses NAD as a cofactor. (**B**) Refolded rSasF following denaturing chromatography from (A) binds NADH with a dissociation constant (*K*_d_) of ∼1 μM as determined fluorometrically. (**C**) ConSurf conservation analysis of rSasF indicating amino acid–level conservation across the top 500 sequenced *S. aureus* strains in the NCBI genome database. (**D**) Experimental scheme for the in vitro epimerization reaction model described in (E) and (F). (**E**) rSasF epimerization of β-GalNAc-MBu results in the production of significantly more MBu when treated with β-*N*-acetylglucosaminidase S compared with the no-rSasF control treated with β-*N*-acetylglucosaminidase. (**F**) Addition of rSasF to α-GalNAc-MBu substrate and subsequent cleavage with α-*N*-acetylgalactosaminidase results in a significantly slower initial reaction rate as determined by the linear reaction rate slope compared with the untreated control. [(E) and (F)] Shown are means ± SD for three biological replicates. Data shown in (A) and (B) are from one replicate from one batch of denatured rSasF. (D) Created in BioRender. T. Enroth (2026), https://BioRender.com/x57711h.

To measure the affinity of NADH toward SasF, we refolded the denatured rSasF using diafiltration against phosphate-buffered saline (PBS) and determined NADH binding fluorometrically, resulting in a dissociation constant of ∼1 μM (Fig. 3B). The SasF affinity for NADH falls within the reported extracellular concentration of NADH found during periods of host stress (*53*, *54*) and within concentrations known to be important for inflammation signaling (*55*). The SasF tertiary structure is unlike that of any known epimerases (Fig. 3C), but there is conservation in the NAD-binding properties of the enzyme.

### Monitoring SasF glycoside epimerization

To investigate SasF epimerization, we developed a new isomerization enzyme assay. We used GalNAc substrate that contains an O-linked 4-methylumbelliferyl (*56*) group attached to the anomeric carbon of GalNAc in the β- orientation (hereafter referred to as β-GalNAc-MBu). If rSasF can isomerize β-GalNAc-MBu to β-GlcNAc-MBu, then product formation can be measured with commercially available *N*-acetylglucosaminidase S (Fig. 3D). rSasF addition to a β-GalNAc-MBu reaction mixture results in significantly increased production of free MBu compared with the no-enzyme control (Fig. 3E and fig. S11). We compared the rSasF enzyme reaction with that of *N*-acetylglucosaminidase S with substrate only, as we observed a small amount of cross reactivity. As predicted, this control did not produce the same amount of free MBu, as was produced in the presence of rSasF. To further use this new enzyme assay, we speculated that rSasF may be able to epimerize the alternative anomer, α-GalNAc-MBu to α-GlcNAc-MBu. Here, and in agreement with this hypothesis, rSasF treatment of α-GalNAc-MBu and the subsequent addition of a commercially available α-*N*-acetylgalactosaminidase resulted in a lower initial linear reaction rate (Fig. 3F), suggesting that rSasF addition to α-GalNAc-MBu results in less GalNAc in the treated sample.

Given that SasF binds NAD and we hypothesize that the epimerization mechanism depends on the cyclic reduction and oxidation of NAD^+^, we hypothesized that rSasF epimerization may be poisoned by NADH addition. Using a colorimetric GalNAc substrate (β-GalNAc-pNA), we observed that the addition of NADH to rSasF significantly decreases the rate of reaction upon *N*-acetylglucosaminidase S addition, with no detectible signal observed until ∼30 min post–*N*-acetylglucosaminidase S addition (fig. S12). Therefore, these data support the hypothesis that rSasF epimerization is dependent NAD^+^ to initiate the epimerization reaction. In all, the in vitro epimerization data support our preliminary NMR observations, suggesting that rSasF can epimerize both the α- and β-GalNAc-MBu anomers and that rSasF activity can be measured effectively in vitro.

### SasF is a MRSA virulence factor

Given that our previous data shows an up-regulation of *sasF* during skin colonization (Fig. 1A), we hypothesized that SasF may be involved in the transition from skin colonization to infection. A report on *S. aureus* surface protein function for skin and soft tissue infection showed a trending decrease in virulence when *sasF* is mutated (*57*). To address this question in our MRSA strain, we performed a skin abscess mouse model and found that the *sasF* mutant abscesses were significantly smaller compared with WT (Fig. 4A). Body weight changes caused by the *sasF* mutant infection were significantly less severe than WT over time (Fig. 4B), suggesting that the *sasF* mutant is impaired in virulence. Last, at 7 days postinfection, we observed significantly less colony-forming units (CFU) found in the abscesses compared with WT (Fig. 4C). The observed changes in skin virulence between the *sasF* mutant and WT strains are not due to hemolysis defects (fig. S13). We observed the *sasF* mutant to be moderately (approximately twofold) more hemolytic to rabbit (fig. S13A) and sheep blood (fig. S13B) cells than WT, which agrees with previous literature implicating cell wall structure to toxin release (*58*). Together, these findings indicated that SasF is an important virulence factor for causing skin infections.

**Fig. 4.**
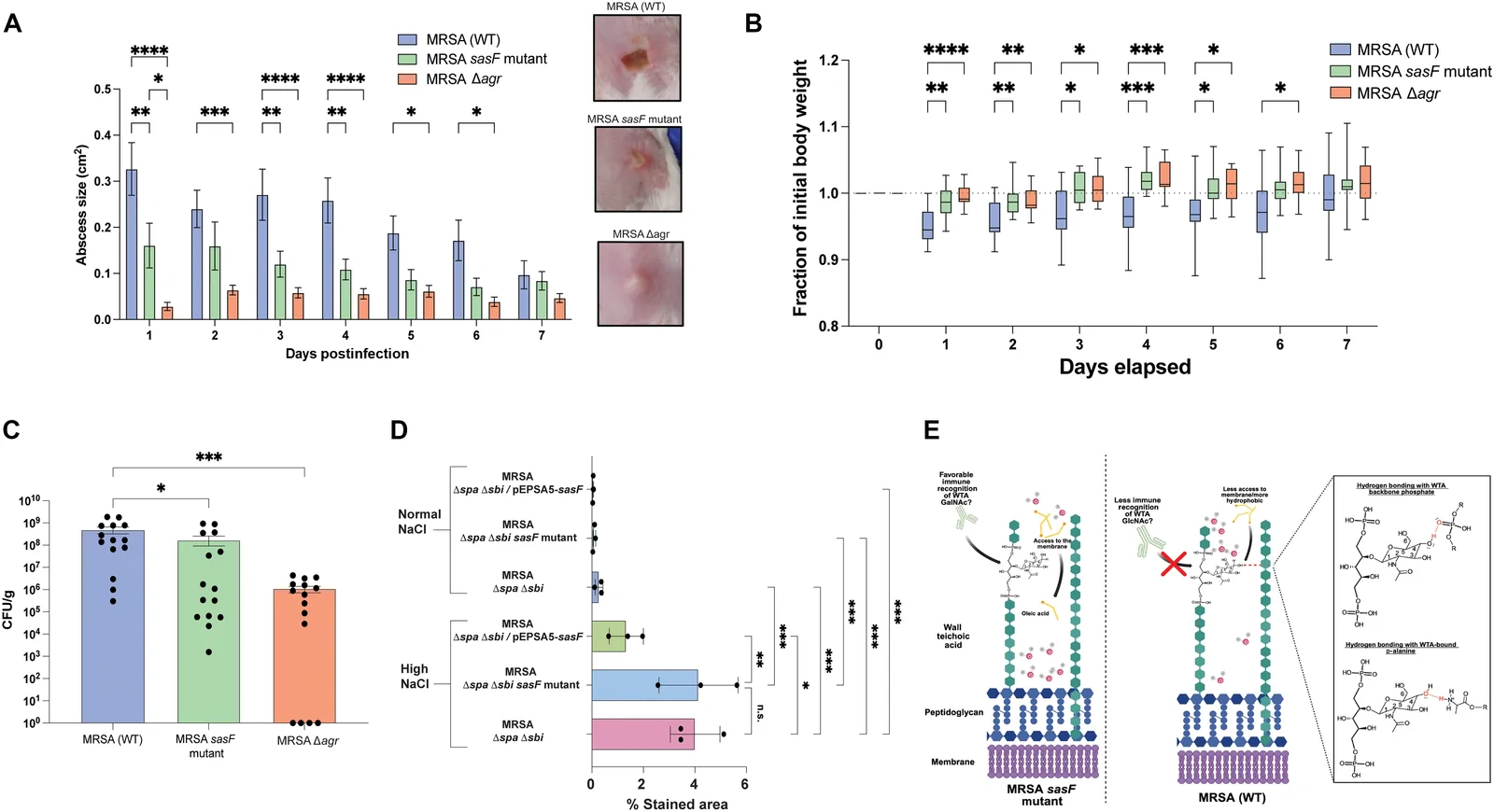
MRSA SasF is crucial for MRSA infection of the skin. (**A**) Quantification and representative photos of the abscess sizes caused by infection with the MRSA *sasF* mutant via ImageJ indicate a significantly smaller abscess size caused by the *sasF* mutant over time compared with MRSA (WT). (**B**) Body weight changes over time (expressed as the fraction of the starting body weight for each mouse) indicate that mice infected with the MRSA *sasF* mutant are less severely affected by inoculation with the MRSA *sasF* mutant and suggest that SasF is a skin virulence factor. (**C**) Abscesses after 7 days contain significantly less CFU/g in mice infected by the MRSA *sasF* mutant compared with MRSA (WT). (**D**) Overexpression of *sasF* during salt stress causes significantly less MGL staining of purified sacculi, suggesting that SasF epimerizes GalNAc at the cell surface. (**E**) Proposed model for the function of SasF based on the data herein. Shown are data from 15 individual mice infected over three independent experiments. For (B) and (D), statistics were performed by a two-way RM-ANOVA, and, for (E), statistics were performed using a nonparametric one-way ANOVA. Shown are mean min to max for B and means ± SEM for (D) and (E). For (D), shown are means ± SD for three biological replicates, and the statistics were performed using a Student’s *t* test. (E) Created in BioRender. T. Enroth (2026), https://BioRender.com/dktazh5.

Healthy human skin and sweat have relatively high NaCl concentrations in the range of 60 to 100 mM (*59*, *60*). In skin diseases like atopic dermatitis (AD), there is a substantial increase in salt concentrations within lesions, up to 80-fold that of healthy skin (*61*). We observed that high NaCl concentrations significantly decrease *sasF* promoter activity (fig. S14A) without affecting growth (fig. S14B), creating a colonization environment where MRSA WTA molecules may become decorated with GalNAc. Certain immune cells such as dendritic cells and macrophages express macrophage galactose-type lectin (MGL) that can bind GalNAc (*62*, *63*) and are known to be enriched in human biopsy samples from patients with AD (*64*). Therefore, we tested whether MGL might recognize MRSA WTA when the cells are grown in high salinity. Although previous work has demonstrated that MGL can recognize GalNAc on WTA of certain *S. aureus* strains within the ST395 lineage (*65*), to our knowledge, MGL has not been shown to recognize USA300 MRSA WTA or similar strains. We observed little staining of MRSA sacculi grown in normal TSB (Fig. 4D), which is in agreement with reports that USA300 strains grown under normal laboratory conditions are not recognized by MGL (*65*), However, when sacculi were prepared from cells grown in high NaCl, we observed significant staining of sacculi by MGL, irrespective of *sasF* being present (Fig. 4D). Sacculi prepared from cells grown in high NaCl and containing a *sasF* overexpression plasmid were stained significantly less with MGL (Fig. 4D). Our data show that, under certain growth conditions, MGL may recognize altered glycosides on MRSA cell surfaces. These data could have potentially broad implications for how *S. aureus* is recognized in certain body sites by host defenses.

## DISCUSSION

The cell wall is the primary interface between a bacterial cell and its external environment. It also acts as a critical barrier against host-derived antibacterial threats and helps maintain a surface environment favorable to bacterial survival. Among the key components of the cell wall are WTAs, sortase-anchored proteins, and lipoteichoic acids that project into the peptidoglycan layer from the membrane. It is well-known that d-alanylation of teichoic acids contributes to resistance against host antimicrobial peptides (*17*). WTAs have also been implicated in helping bacteria resist host stressors such as fatty acids (*19*), although the underlying mechanisms behind this remain poorly understood. Here, we describe a new enzymatic role for SasF as an epimerase that is crucial for maintaining the structure of WTA for protection against innate defense components of the skin.

*S. aureus* WTA molecules from USA300 (ST8) and similar lineages have been reported to be decorated exclusively with either α- or β-GlcNAc (*14*, *15*). However, using in vitro reactions to study the glycosyltransferase that adds β-GlcNAc to WTA (TarS), the Walker group observed that UDP-GalNAc can be used as a substrate for TarS, albeit at a lower efficiency than UDP-GlcNAc (*14*). TarM glycosyltransferase, which adds α-GlcNAc to WTA, might potentially also use UDP-GalNAc at a low efficiency, although now the TarM substrate preference is unknown. Seeing that TarS can, at least to a certain degree, use UDP-GalNAc as a substrate, *S. aureus* may have mechanisms like SasF for preventing the accumulation of GalNAc onto WTA molecules. This could also be important for scenarios when the intracellular pools of UDP-GlcNAc shift to UDP-GalNAc. On the other hand, there may be environmental scenarios where *S. aureus* WTA decoration with GalNAc is preferred to GlcNAc. Either way, SasF’s role in WTA homeostasis has been overlooked because all previous WTA structural analyses were from SasF functional strains. Although our data support a role for SasF in facilitating epimerization, further biochemical experiments should be performed to confirm that the epimerization is occurring at C4 and not another carbohydrate atom.

There is one *S. aureus* strain called PS187 that decorates its WTA exclusively with GalNAc despite SasF being present (*66*). However, PS187 has a structurally dissimilar WTA backbone composed of glycerol phosphate, like that of *Staphylococcus epidermidis*, instead of the ribitol phosphate backbone used by most *S. aureus* lineages. Sequenced *S. epidermidis* isolates also encode a SasF homolog that is 35 to 40% conserved to *S. aureus* SasF (fig. S15A). When we compared protein structures, the predicted structure for SasF from USA300 LAC and the *S. epidermidis* homolog are poorly conserved, especially outside the core globular regions of the protein [root mean square deviation (RMSD), 4.315] (fig. S15B). SasF might require specific residue contacts to discriminate between the glycerol versus ribitol WTA backbone to function; however, this will require future investigation.

While more structural studies will be needed to determine the exact SasF substrate specificity, there are considerations for the *S. aureus* field if SasF is really a WTA glycoside epimerase. The structural differences between other WTA glycosides and WTA-GlcNAc seem small, but epimerizing a carbohydrate would likely result in broad WTA structural and biochemical changes (Fig. 4E). First, the WTA polymers might be more sterically repelled from one another if GalNAc were epimerized to GlcNAc given that epimerization happens without glycosidic cleavage. There could also be differences in the glycoside accessibility to free water, which may alter extracellular water activity, and could explain surface hydrophilicity differences in the *sasF* mutant compared with WT (Fig. 1D). Further, we observed a modest increase in mCRAMP susceptibility for the *sasF* mutant (fig. S5) that could be explained by general steric changes the cell wall. In agreement with this, we observed that the *sasF* mutant is more resistant to the negatively charged dye Congo red compared with WT (fig. S3). However, binding of positively charged cytochrome C was not different between the *sasF* mutant and WT (Fig. 1E). Therefore, SasF is not affecting MRSA surface charge, but rather susceptibility differences to various stressors potentially due to the steric differences of WTA bound GalNAc versus GlcNAc. These results may be explained by differences in GalNAc versus GlcNAc capacity to hydrogen bond with neighboring WTA molecules on the cell wall (Fig. 4E). It is possible that the equatorially arranged hydroxyl group of GlcNAc is able to hydrogen bond with either the backbone phosphates or neighboring d-alanine residues. This bonding may promote structural differences in the WTA molecules and changes in the general cell surface properties that we observed herein; however, each of these hypotheses should be explored further. However, these hypotheses may help explain why WTA molecules of *S. aureus* are nearly exclusively decorated with GlcNAc. In addition to this, our data suggest that cell surface maintenance by SasF is crucial for virulence in a skin abscess model, implying that not only does the skin environment pose a distinct challenge toward the bacterial cell but also that the cell wall is a crucial first line of defense against host antimicrobial stressors (Fig. 4E).

We observed that SasF may bind NAD at concentrations known to be physiologically relevant during infection and inflammation (*53*, *54*) in a similar manner to known epimerases. Upon secretion of SasF, the protein would likely be free of bound NAD as it is being surface localized. Given that without SasF the bacteria are more cytotoxic (fig. S12), increased lysis of host cells may free NAD enabling SasF binding, at least during initial stages of infection. However, in an established, inflammatory infection, there is a large concentration of free NAD, and SasF will be able to bind NAD soon after secretion.

To our knowledge, SasF is the first identified surface epimerase in *S. aureus*. While SasF is important for responses to environmental stressors, the enzyme activity is likely applicable more broadly. For instance, phage therapy has received revitalized interest in recent years as conventional antibiotics lose efficacy, and the biomedical field turns to new alternatives. Seeing that SasF modifies *S. aureus* phage infection potential, it is compelling to consider the protein as a probable synergistic target alongside the application of phages. For example, the lytic phage ɸP68 may use the changed WTA structure of the *sasF* mutant as a receptor (Fig. 1J). Therefore, by inhibiting SasF and applying lytic phages such as ɸP68, *S. aureus* infections might be able to be effectively treated using phage therapy. Thus, considering SasF as a target toward modulating phage susceptibility might broaden the consideration of phage therapy as an alternative treatment strategy for MRSA.

Similarly, our findings may have implications on the efficacy of vaccines against *S. aureus* that have failed to be efficacious. WTA molecules and their glycosidic decorations are known to be a predominant surface antigen of *S. aureus* (*67*, *68*), but antibody-based vaccines against *S. aureus* have thus far failed (*69**–**72*). We determined that when *S. aureus* is in common body sites with high salt, SasF will not be expressed and alternative glycosides will accumulate on the surface (Fig. 4D), which was not previously observed in normal lab growth conditions for *S. aureus* (*14*, *15*). Considering our findings, the inclusion of other, non-GlcNAc WTA epitopes in vaccine formulations could improve vaccine effectiveness against *S. aureus*. Although our data in Fig. 2 suggest that there may be GalNAc molecules decorating the WTA polymers of the *sasF* mutant, further NMR experiments will be needed to corroborate findings. The chemical shifts observed using both WTA extracted from the *sasF* mutant and the isopropyl glycosides are consistent with the mutant WTA being decorated with GalNAc and SasF being involved in glycoside epimerization. This work does not rule out a role for SasF in epimerizing glycosides in other contexts, as our data focuses on a role for SasF in maintaining WTA glycoside structures, perhaps in metabolic contexts or those involved with other extracellular polysaccharides.

Together, our goal in this study was to mechanistically define a function for *S. aureus* SasF, a new sortase-anchored epimerase that is up-regulated in skin conditions. Beyond *S. aureus*, homologs of SasF are encoded by a variety of commensal Staphylococci that include *S. epidermidis*, *Staphylococcus simulans*, *Staphylococcus capitis*, *Staphylococcus hominis*, and *Staphylococcus haemolyticus*, all of which are part of the human skin microbiome (*73*) and can sometimes cause disease. Homologs of the protein are also found in Staphylococci known to colonize and infect animals such as *Staphylococcus pseudintermedius* (*74*), *Staphylococcus felis* (*75*), and *Staphylococcus agnetis* (*76*). Thus, the maintenance of the bacterial cell surface glycoside composition appears to be a conserved challenge that warrants the conservation of SasF for ensuring GlcNAc is displayed on the surface. This may be because non-GlcNAc glycosides can be recognized by host immune receptors or potentially the maintenance of the proper level of cell surface hydrophobicity. Beyond Staphylococci, the implications of SasF in maintaining the WTA structure remain less clear as WTA polymer structures vary considerably in terms of chemistry and carbohydrate composition. However, given that there are no SasF homologs present in human or animal proteomes, our work reveals SasF as an exciting *S. aureus*/MRSA therapeutic target given its high conservation and implication in virulence.

## MATERIALS AND METHODS

### Strains and growth conditions

All strains used are listed in table S1. Unless otherwise specified, all *S. aureus* stains grown overnight were done so in TSB (BD, ref. 211822) in a 5-ml volume using sterile 20× 150 mM borosilicate glass tubes (Thermo Fisher Scientific, ref. 14-961-33) shaking at 220 rpm at 37°C, all inoculated from a glycerol stock stored at −80°C. *Escherichia coli* strains were grown similarly in LB broth (RPI, ref. L24066). If bacteria were grown on agar medium, TSA (BD, ref. 230920) was used for *S. aureus* and LBA (RPI, ref. L24020) was used for *E. coli*. If using antibiotics to select for mutants or to maintain plasmids, chloramphenicol was used at 10 μg/ml (Thermo Fisher Scientific, ref. AAB2084114) in 200 proof ethanol, ampicillin was used at 100 μg/ml in H_2_O (RPI, ref., A40040) and filter sterilized before use, carbenicillin was used at 100 μg/ml in H_2_O (Thermo Fisher Scientific, ref. J61949.06) and filter sterilized before use, and erythromycin was used at 10 μg/ml (RPI, ref. E57000) in 200 proof ethanol.

### Strain construction and phage transduction

Various *S. aureus* mutants in this study were created by back-crossing transposon mutants from the Nebraska Transposon Mutant Library (*77*, *78*) into the WT AH1263 background using phage transduction. Plasmids were also transferred to recipient *S. aureus* strains by phage transduction after initial electroporation of the plasmid into *S. aureus* RN4220. To do so, a ɸ11 phage lysate was generated on the donor strain of interest (containing the transposon of interest or plasmid) by adding 250 μl of a high-titer filtered ɸ11 lysate to the donor strain adjusted to an OD_600_ of 0.2 in 10 ml of a 50/50 mixture of TSB and phage lysis buffer. The phage lysis buffer used contains (per liter) 250 mg of MgSO_4_, 590 mg of CaCl_2_, 6.06 g of tris-HCl (pH 7.8), 5.9 g of NaCl, and 1 g of gelatin, which were all dissolved in Milli-Q water and autoclaved. The phage solution was incubated overnight at RT until the solution cleared, indicating sufficient lysis of the bacterial strain. All phage lysates were filter sterilized using a 0.22-μm filter unit before use. To transduce using the above-prepared phage lysate, 1 ml of an overnight culture of the recipient strain was added to four individual sterile 15-ml conical tubes. CaCl_2_ was added to each 1 ml of aliquot to a final concentration of 12 mM. Then, 1 ml of the above phage lysate was added to one recipient strain aliquot, 100 μl was added to another, 10 μl was added to the third aliquot, and one recipient strain was reserved as a no-phage control. The phage-bacteria mixtures were incubated for 15 min at 37°C, after which 2 ml of sodium citrate blocking solution (1.7 ml of sterile 1 M sodium citrate and 48.3 ml of sterile Milli-Q) was added to each tube and mixed by hand briefly. The samples were centrifuged at 4000*g* for 10 min, and the supernatant was decanted. The pellets were resuspended in 2 ml of a 50/50 mixture of TSB/sodium citrate blocking solution and allowed to incubate for 1 hour at 37°C. After an hour, the samples were centrifuged as above, resuspended in 1 ml of the 50/50 mixture of TSB/sodium citrate blocking solution, and plated on TSB containing the appropriate antibiotic and 20 mM sodium citrate. The plates were incubated inverted overnight at 37°C at which point colonies were patched on a fresh TSB and 20 mM sodium citrate plate and used for colony polymerase chain reaction (PCR) screening of the proper mutation/plasmid.

### PCR screening for proper mutations or plasmids and sequence confirmations

After transductions or transformations and before using any of the plasmids constructed in this study, colony PCRs were performed to check transposon integration into the genome of recipient strains or the presence of the desired plasmid using plasmids that flank the insertion or gene. For all plasmids used, whole-plasmid sequencing (using NanoPore sequencing) was performed from plasmids miniprepped from *E. coli* using the QIAprep spin Miniprep Kit (QIAGEN, ref. 27106). The presence of whole-plasmid sequence–confirmed plasmids transduced into *S. aureus* was confirmed once more by colony PCR before use of the strain. All primers used in this study are listed in table S1.

### *sasF* complementation vector construction

A *sasF* complementation vector was created via Gibson assembly. pCM28 was miniprepped from AH4532 (*E. coli* DC10B). The plasmid was then digested using Bam HI–HF (NEB, ref. R3136S) and Pst I–HF (NEB, ref. R3140S) and subsequently PCR purified using the QIAGEN QIAquick PCR purification kit (QIAGEN, ref. 28106). The *sasF* ORF, native promoter, and terminator were amplified from AH1263 gDNA using Q5 DNA polymerase and the primers SasFComp_F and SasFComp_R. The PCR product was PCR purified, combined with the digested pCM28, and ligated using the NEBuilder HiFi DNA Assembly Cloning Kit (NEB, ref. E5520S) according to the manufacturer’s instructions. The assembly mix was then used to transform *E. coli* DH5α, after which colonies were obtained, PCR confirmed to contain the correct plasmid, miniprepped, and whole-plasmid sequenced. Plasmid with the correct sequence was then used to transform *S. aureus* RN4220 and transferred to the *sasF* mutant by phage transduction as above.

### *sasF* overexpression vector construction

*sasF* was overexpressed using the xylose-inducible plasmid pEPSA5. To construct this plasmid, pEPSA5 was miniprepped from AH0222 (*E. coli* DH5α). The plasmid was digested using Bam HI–HF (NEB, ref. R3136S) and Sac I–HF (NEB, ref. R3156S) and PCR purified as above. The *sasF* ORF without its native promoter was amplified using Q5 DNA polymerase (NEB, ref. M0491S) and the primers SasF_SacI_F and SasF_BamHI_R. The PCR product was purified and digested with Sac I–HF and Bam HI–HF according to the manufacturer’s suggestions and then PCR purified once more. The digested plasmid and PCR product were ligated using T4 DNA ligase (NEB, ref. M020S) according to the manufacturer’s recommendations. The plasmid was used to transform *E. coli* DH5α, screened, and sequenced as above.

### P*sasF* promoter-GFP fusion construct construction

The SasF promoter was amplified by PCR using PsasF_XbaI_F and PsasF_KpnI_R and Q5 polymerase. The amplicon was PCR purified and subsequently digested using Xba I and Kpn I. pCM29 was miniprepped from DH5α and digested with the Xba I and Kpn I. Both digestion products were PCR purified and then ligated together using T4 DNA ligase (NEB, ref. M0202S). Chemically competent DH5α cells were transformed with the ligation mixture, colonies were patched and confirmed by PCR to contain the correct plasmid, and the plasmid was miniprepped and sequence confirmed. The confirmed plasmid was then transformed directly into AH1263, patched, and PCR confirmed, and correct transformants were used as the WT *sasF* promoter-fusion strain where sGFP transcription is driven off the *sasF* promoter.

### rSasF protein expression

Proteins were all purified from the *E. coli* protein expression strain ER2566 using the expression vector pTEV5 (*79*). All proteins purified contain an N-terminal 6X histidine tag that is separated from the protein-encoded region by a tobacco etch virus (TEV) cleavage site; however, all protein constructs used contain an intact 6X histidine tag. Upon piloting protein purification initially, we experienced severe protein aggregate formation using the full-length SasF protein caused by the N-terminal helical bundle (Fig. 1C), which prevented the purification of full-length SasF using our expression and purification schemes. Further, we purified the protein without the C-terminal sortase signal and anchor. Thus, all protein constructs purified include only the core globular portion of the protein, where the rSasF protein purified starts at threonine-113 and ends at arginine-537. Bacterial expression strains were grown overnight in 50 ml of LB containing carbenicillin (100 μg/ml). Ten milliliters of this overnight culture was added to 1 liter of sterile LB containing carbenicillin (100 μg/ml) in a 4-liter flask and allowed to incubate at 37°C shaking 210 rpm until mid-log was reached [optical density at 600 nm (OD_600_) of approximately equal to 0.6]. Once the cultures reached mid-log, isopropyl-β-d-1-thiogalactopyranoside was added to a final concentration of 1 mM and allowed to induce for 4 hours at 37°C shaking 210 rpm. After induction, the cells were pelleted at 4000*g* at 4°C for 30 min. The supernatant was decanted, and the pellets were stored at 20°C overnight.

### rSasF protein purification by FPLC

All protein was purified using either a GE Healthcare AKTA Explorer 10 or a Bio-Rad NGC Scout Plus fast protein liquid chromatography (FPLC) system. The pellets from 4 liters total of the spun down cultures (above) were resuspended in 100 ml of equilibration buffer [50 mM sodium phosphate (pH 8.0) with 300 μM NaCl and 20 mM imidazole] containing two EDTA-free protease inhibitor cocktail tablets (RPI, ref. P52370) and 1.1× FastBreak cell lysis reagent (Promega, ref. V8573) and allowed to incubate on ice for 30 min. After 30 min, the cells were manually extruded through a 23-gauge needle twice. After extrusion, the cell lysate was pelleted at 17,000*g* for 35 min at 4°C, and the supernatant was collected and filtered through a 0.22-μm vacuum filtration unit. The filtered lysate was then passed over an equilibrated [using 50 mM sodium phosphate (pH 8.0) with 300 μM NaCl and 20 mM imidazole] 5-ml column volume HisTrap HP or HisTrap HF (depending on the protein batch) nickel affinity chromatography column. The column was washed sufficiently after application of protein and eluted using a shallow gradient of increasing percentage of elution buffer [50 mM sodium phosphate (pH 8.0), 300 μM NaCl, and 250 mM imidazole]. Fractions within the probable eluted protein fractions were checked for purity and desired protein presence by SDS–polyacrylamide gel electrophoresis and staining with InstantBlue Coomassie stain (Abcam, ref. ab119211). Fractions were then pooled and concentrated using an Amicon Ultra-15 3K molecular weight cutoff (MWCO) concentrator (Sigma-Aldrich, ref. UFC900324) unit by centrifugation at 4000*g* at 4°C. Concentrated protein was added to a presoaked dialysis bag (Spectra/Por, ref. 132665) with a MWCO of 6 to 8 kDa. The protein in the dialysis bag was then dialyzed against 4 liters of 1× DPBS (Corning, ref. 20-031-CV) three times, twice for 4 hours, and once overnight, all at 4°C. The dialyzed protein was then concentrated again using an Amicon Ultra-15 3K MWCO filter if needed and then flash frozen as 20 μl of beads in liquid nitrogen. The frozen protein beads were collected and stored at −80°C until use. All protein concentrations were subsequently measured using a Pierce BCA protein assay kit (Thermo Fisher Scientific, ref. 23225) according to the manufacturer’s suggested protocol. A Coomassie-stained gel of a dilution series of rSasF along with the protein sequence as purified is provided in fig. S16.

### Anti-SasF polyclonal antiserum development

SasF lacking the N-terminal three-helix bundle was purified as described above and concentrated to 13 mg/ml before sending 200 μl of protein sample to Pacific Immunology (San Diego, California, USA). Two rabbits were immunized four times with the protein samples along with AdjuLite adjuvant. Four bleeds were obtained per animal and used as anti-SasF antisera for Western blots against SasF.

### Promoter activity and Western blot analyses of SasF

The AH1263 pCM29::P*sasF* promoter-fusion experiment was used in all experiments that correlate promoter activity to protein production. Here, the reporter strain was diluted 1:100 in 5 ml of TSB with oleic acid added to the medium to the indicated concentrations (or a solvent-only control). Oleic acid (Thermo Fisher Scientific, ref. 11-101-2506) was first prepared as a 100 mM stock in 200 proof ethanol before adding the fatty acid to TSB. The 1:100-diluted reporter strain (200 μl) was then added to a sterile black-walled 96-well plate. The plate was incubated at 37°C shaking at 800 rpm on a Stuart microtiter plate reader. Every hour for 7 hours, the plate was measured on a Tecan infinite 200 pro microplate reader for both bacterial density at OD_600_ and for fluorescence using a 492-nm excitation wavelength, a 539-nm emission wavelength, a 9-nm excitation bandwidth, and a 20-nm emission bandwidth. For all fluorescence readings, the gain was set to 60 with 10 flashes, a 20-μs integration time, and a 20,600-μm *Z*-position. At 4 and 7 hours, 200-μl samples from each biological triplicate were obtained for Western blot analyses of SasF protein abundance. Here, the 200-μl sample was spun down, and the cells were resuspended in 50 μl of sterile PBS. Three microliters of lysostaphin (10 mg/ml; Ambi Products LLC, ref. LSPN-50) in 20 mM sodium acetate (pH 4.5) was added to lyse the cells, and the samples were incubated at 37°C until the cells were lysed. One hundred fifty microliters of 4× Laemmli sample buffer (Bio-Rad, ref. 1610747) was added and mixed thoroughly. The samples in sample buffer were kept at −20°C until use. For control Western blots, 200 μl of overnight culture was obtained and prepared the same way as above. Before separation by gel electrophoresis, the samples were boiled at 95°C for 10 min, and 23.5 μl of the samples were added to each well of a 4 to 20% mini-PROTEAN TGX precast gel (Bio-Rad, ref. 4568093) alongside a Precision Plus Protein Kaleidoscope ladder (Bio-Rad, ref. 1610375). The gels were run at 150 V and immediately transferred onto a Trans-Blot Turbo Mini 0.2-μm polyvinylidene difluoride membrane (Bio-Rad, ref. 1704156) using a Bio-Rad Trans-Blot Turbo transfer system using the high–molecular weight setting. Following transfer, the membrane was cut at ∼55 kDa to prevent antibody sequestration by the *S. aureus* immunoglobulin binding proteins Spa and Sbi, and the two membrane halves were stained in parallel using the same concentrations of antibodies. The membranes were blocked for 1 hour at RT using Licor Intercept TBS blocking buffer (Licorbio, ref. 927-60001). Anti-SasF polyclonal antiserum was added at 1:250 in Licor Intercept TBS blocking buffer and allowed to incubate rocking at RT for 1 hour. After 1 hour, the membranes were washed with 1× TBS-T three times for 5 min each, and goat anti-rabbit IRdye 800 secondary antibody (Licorbio, ref. 926–32211) was added at 1:12,000 in Licor Intercept TBS blocking buffer and allowed to incubate for an hour at RT with rocking. The membranes were then washed once more as above and immediately imaged on a Licor Odyssey CLx.

### Oleic acid susceptibility assay

The oleic acid susceptibility assays were adapted from previous studies (*13*, *80*). Briefly, 1 ml of an overnight culture of each strain of interest was pelleted at 13,000*g* for 1 min, washed once in sterile PBS (Thermo Fisher Scientific, ref. BP2944100), and adjusted to an OD_600_ of 1.0 in PBS. The normalized strains were then serially diluted 10-fold from undilute to 10^−7^ in sterile PBS. Each serial dilution (5 μl) for each strain was spotted onto BHI medium containing either 3 mM oleic acid or an equal volume of 200 proof ethanol as the solvent control and allowed to dry. To prepare the oleic acid plates, a 1 M oleic acid stock was made in 200 proof ethanol. Then, 75 μl of the 1 M stock (or solvent only control) was added quickly to 25 ml of molten BHI agar that is cool enough to be held comfortably in the hand. The medium with oleic acid was inverted manually six times before pouring the solution into a sterile square petri dish. The plates were allowed to solidify and dry for ∼30 min before plating the bacteria on the medium. All plates were inverted after spotting of bacteria and incubated overnight at 37°C before imaging.

### Tunicamycin and tarocin A1 sensitivity assays

The tunicamycin and tarocin A1 susceptibility assay were performed essentially as the oleic acid susceptibility assays were, with a few modifications. Tunicamycin (Thermo Fisher Scientific, ref. AAJ62217MA) and tarocin A1 (Sigma-Aldrich, ref. SML1677) were resuspended to 5 mg/ml in 100% dimethyl sulfoxide (DMSO). Tunicamycin or tarocin A1 (or an equal volume of DMSO as a solvent-only control) was added to 25 ml of molten TSA to a final concentration of 2 μg/ml and hand mixed by inversion six times. The media were allowed to solidify and dry in the dark before spotting of bacteria. An overnight culture (1 ml) of each strain of interest was pelleted at 13,000*g* for 1 min, washed once, and then adjusted to an OD_600_ of 1.0 in PBS. The normalized strains were then serially diluted 10-fold from undilute to 10^−7^ in sterile PBS. Each serial dilution (4 μl) was spotted on the plates and allowed to dry. The plates were then inverted and incubated at 37°C overnight before imaging.

### Congo red susceptibility assay

Congo red analyses (CFU/ml) and bacteria preparation were performed as described above for the tunicamycin and tarocin A1 sensitivity assays, except Congo red (Thermo Fisher Scientific, ref. AAB2431022) was added to 0.18% (w/v) into molten TSA instead of tunicamycin or tarocin A1.

### Cell surface hydrophilicity assay

The cell surface hydrophilicity was assessed exactly as was previously described (*13*) using *n*-hexadecane (Thermo Fisher Scientific, ref. AAA10322AP) as the hydrophobic partitioning agent.

### mCRAMP susceptibility assay

An overnight culture (1 ml) was spun down and washed once in 10 mM phosphate buffer (pH 7.4). The strains were normalized to ∼1 × 10^9^ CFU/ml in 1 ml of 10 mM phosphate buffer (pH 7.4). Synthetic mCRAMP (AnaSpec, ref. AS-61305) was resuspended to 40 μM in 10 mM phosphate buffer (pH 7.4). To a clear flat bottomed 96-well plate, 198 μl of the 40 μM mCRAMP was added to each well, and, then, 2 μl of the 1 × 10^9^ CFU/ml solutions of each strain was added to the wells with mCRAMP. This equates to a starting bacterial concentration added of 1 × 10^7^ CFU/ml. Immediately, 20 μl of the solution was serially diluted in sterile PBS and plated for CFU enumeration on TSA. The 96-well plate was plate was placed at 37°C in a Stuart microtiter plate incubator shaking at 800 rpm. For each time point, 20 μl of sample was obtained for serial dilution and enumeration (CFU/ml), where 20 μl of each serial dilution was dripped on a sterile TSA plate, allowed to dry, inverted, and incubated at 37°C overnight.

### Skin colonization IVIS

Skin colonization experiments using IVIS in vivo bioluminescent imaging (PerkinElmer IVIS Spectrum/CT) were performed exactly as described previously, using a total inoculum of 10^8^ CFU per site (*7*). The strains used were constitutively luminescent strains of either MRSA (WT) or the *sasF* mutant containing a chromosomally integrated pHC125 vector, as described previously (*81*).

### Matrix adhesion assays

Purified fibrinogen (Sigma-Aldrich, ref. F3879) and fibronectin (EMD Millipore, ref. 341635) were resuspended to a concentration of 20 μg/ml in PBS. Before the adhesion assay, a 96-well tissue culture–treated plate was incubated with 50 μl of either the fibrinogen or fibronectin stocks (20 μg/ml) overnight at 4°C. On the day of the assay, overnight cultures of the strains of interest were subcultured 1:100 in 10 ml of sterile TSB in a 50-ml Erlenmeyer flask and allowed to incubate at 37°C shaking 220 rpm until they reached mid-log (OD_600_ of ∼0.5 to 0.6). The cultures were pelleted and resuspended in 1 ml of sterile PBS. Each culture was normalized to OD_600_ of 1.0 in 2 ml of sterile PBS. The plates incubated with the above matrix components were washed three times with 100 μl of PBS per well. One hundred fifty microliters of 5% bovine serum albumin (BSA; Thermo Fisher Scientific, ref. BP9703100) in PBS was added per well and allowed to incubate at 37°C for 2 hours. The plate was washed three times with 200 μl of PBS, and 100 μl of the normalized bacterial culture was added to their appropriate wells. The bacteria were allowed to adhere for 1 hour at 37°C, after which the wells were gently washed with 150 μl of PBS. The plate was allowed to dry for 2 hours at 60°C, at which point 80 μl of a 0.1% crystal violet in sterile Milli-Q was added to each well and allowed to incubate for 5 min. The plate was washed under tap water and dried. Thirty-three percent acetic acid (80 μl per well) in Milli-Q was added to each well to dissolve the crystal violet, and the OD_570_ was measured alongside a no-bacteria control blank, which was subtracted from the experimental measurements.

### N/TERT-2G adherence assay

Bacterial adherence to differentiated N/TERT-2G cells was assessed as was done previously with modifications (*26*). Here, the N/TERT-2G cells were grown and differentiated on transwells to better replicate human stratified dermal layer. Warm DFK medium (900 μl) was added to each well into which the transwells would be placed. N/TERT-2G cells were adjusted to 10^5^ cells/ml in experimental medium, and, then, 250 μl of the cell suspension was pipetted into each transwell. The seeded transwells were incubated overnight at 37°C with a 5% CO_2_ atmosphere. The basal medium outside the transwell was removed and replaced with fresh, warm experimental medium. Then, the liquid in each transwell was aspirated carefully and disposed. Replacement of the basal medium and aspiration of the liquid from each transwell was performed for a total of 7 days to achieve a differentiated layer of N/TERT-2G cells, which is used as a model for bacterial adherence to the stratum corneum. At 7 days, there was no liquid present above the cells growing on the transwell, which was used as a marker for sufficient differentiation. On the day of the adherence assay, the bacteria were grown, prepped, and used in an adherence assay, as was described previously (*26*).

### Crystal violet biomass/biofilm formation assay

MRSA *sasF* mutant biomass/biofilm formation was adapted from a previous protocol. Briefly, 250 μl of 20% human plasma (Sigma-Aldrich, ref. P9523) in carbonate/bicarbonate buffer (Sigma-Aldrich, ref. C3041) was added to a 24-well plate and allowed to incubate overnight at 4°C. Overnight cultures of the strains of interest were grown in TSB containing 0.5% (w/v) glucose and 3.0% NaCl as has been described previously. The next day, the plasma was aspirated from each well carefully. The overnight cultures were diluted 1:50 in fresh TSB containing 0.5% (w/v) glucose and 3.0% NaCl, and 400 μl of the dilute bacteria was added per plasma-coated well. The inoculated plate was incubated at 37°C statically for 24 hours. After incubation, the supernatant from each well was carefully aspirated, and each well was washed once with 400 μl of sterile PBS. The plate was baked at 60°C for 1 hour, and 400 μl of 0.05% aqueous crystal violet was added to each well for 5 min and then aspirated. The wells were then washed three times with 400 μl of PBS, and the PBS was aspirated after the third wash. The crystal violet was then dissolved using 400 μl of 30% acetic acid in Milli-Q, and the OD_595_ of the dissolved crystal violet was measured using a Tecan microtiter plate reader. In cases where the absorbance was too high to read, the samples were diluted 1:100 in PBS, and the OD_595_ of the dilute samples was measured.

### Cytochrome C binding analysis

Susceptibility of our strains of interest to cytochrome C (Sigma-Aldrich, ref. C2506; resuspended to 100 mg/ml in PBS) was performed as performed previously (*82*), except that the bacteria were normalized to an OD_600_ of 4.0.

### Phage plaque assays

The bacterial strain indicated (1 ml) was spun down, washed once with PBS, and resuspended to an OD_600_ of 1.0 in PBS. The suspension (50 μl) was combined with 10 ml of 0.5% TSA top agar containing 5 mM CaCl_2_, mixed, and then overlaid onto normal TSA plates supplemented with 5 mM CaCl_2_. The top agar layers were allowed to solidify, at which point 4-μl spots of the indicated serial dilutions for the bacteriophages were carefully pipetted on top of the solidified soft agar lawn. For experiments where rSasF was added before plaque assay, rSasF was added to a final concentration of 15 μM in 1 ml of bacteria normalized to OD_600_ of 1.0 and allowed to incubate for 1 hour rotating at 37°C before adding 100 μl of the treated bacteria to 10 ml of soft agar as above. The spots were allowed to dry, at which point the plates were placed at 37°C uninverted overnight before imaging. All phages used were propagated as above using either the AH1263 as a host for ɸ11 or ɸK or the AH6491 for ɸP68.

### ^13^C-enriched WTA extraction

WTA extractions were adapted from previously described methods with multiple changes (*83*). All strains from which WTA were extracted were grown in sterile and disposable 125-ml Erlenmeyer flasks (Greiner, ref. 679501) in a total volume of 20 ml. ^13^C-enriched TSB was used by adding d-glucose-^13^C_6_ (2.5 g/liter; Sigma-Aldrich, ref. 289374) to TSB without dextrose (BD, ref. 286220). Overnight cultures of the bacterial strains of interest grown in the above medium were pelleted at 4000*g* for 10 min, and the supernatant was decanted. The pellets were washed in 30 ml of 50 mM MES (pH 6.5) and pelleted as above. The supernatant was decanted, and the pellet was resuspended in 30 ml of 50 mM MES (pH 6.5) containing 4% (w/v) SDS. The samples were then added to a water bath at a rolling boil and allowed to boil for 1 hour. The tubes were removed from the boiling water and allowed to cool for 30 min before centrifuging as above. The supernatant was decanted, and the pellet was resuspended in 1 ml of 50 mM MES (pH 6.5). The samples were centrifuged for 2 min at 13,000*g*, and the supernatant was removed. The pellet was resuspended in 1 ml of 50 mM MES (pH 6.5) containing 4% (w/v) SDS and washed another three times as above. After the washes with 50 mM MES (pH 6.5) containing 4% (w/v) SDS, the samples were washed in 50 mM MES (pH 6.5) containing 2% (w/v) NaCl and centrifuged as above. Then, the samples were washed one more time in 50 mM MES (pH 6.5), with the pellets subsequently resuspended in 20 mM tris-HCl (pH 8.0) containing 0.5% (w/v) SDS. Proteinase K (Thermo Fisher Scientific, ref. FERE0491) was added to a final concentration of 80 μg/ml and allowed to incubate at 50°C overnight. The samples were then incubated at 95°C for 10 min to inactivate some of the proteinase K and were then centrifuged at 13,000*g* for 2 min. The supernatant was removed, and the samples were washed four times with sterile Milli-Q water. The washed samples were then resuspended in 1 ml of a freshly made 5% trichloroacetic acid solution in Milli-Q and incubated at 65°C for 4 hours. After 4 hours, the samples were centrifuged for 2 min at 13,000*g*, and the supernatant was divided into two 500 μl of aliquots in new microcentrifuge tubes. The 500-μl samples were then flash frozen in a dry ice-ethanol bath and then lyophilized using a Labconco CentriVap cold trap and a Precision D150 vacuum pump (150 i/m) overnight. The lyophilized WTA samples were stored in an airtight container containing DRIERITE desiccant stones at −20°C until use.

### HSQC analyses of WTA with enzyme addition

All NMR experiments were conducted on Bruker 900 spectrometer equipped with a cryo-probe at 25°C. All NMR data were processed and analyzed using nmrDraw and ccpNMR (*84*, *85*). For uniformly ^13^C-labeled WTA, 2D ^1^H-^13^C HSQC spectra were acquired using eight scans, with 200 increments in the ^13^C dimension and 2048 points in the ^1^H (direct) dimension. WTA samples were resuspended in 300 mM phosphate buffer (pH 7.0) with 5% D_2_O. For enzyme addition experiments, rSasF was added to a final concentration of 10 μM for the time specified in each figure legend. All spectra are decoupled.

### HSQC analyses of isopropyl glycoside WTA mimics and appropriate controls with enzyme addition

All synthetic isopropyl glycoside samples [α-isopropyl GlcNAc (1Click Chemistry, ref. 9C823819) and β-isopropyl GlcNAc (Sigma-Aldrich, ref. ATEH98884953)], the isopropanol control, and the GalNAc control were prepared in 100 mM MES (pH 6.5) with 150 mM NaCl added with 5% D_2_O. The concentration of each compound analyzed was 20 mM to allow for detection of naturally abundant ^13^C. In protein addition experiments, unlabeled rSasF was added to final concentration of 53 μM after the acquisition of an initial untreated spectrum. 2D ^1^H-^13^C HSQC spectra were acquired with 144 scans with 200 increments in carbon and 2048 points in the direct dimension to compensate for lower sensitivity. All spectra are decoupled.

### In vitro epimerization assay

Either the β-GalNAc-MBu anomer (TCI, ref. M3029) or the α-GalNAc-MBu anomer (LGC Standards, ref. TRC-M333500) was resuspended to 1 mM in 1× dPBS (pH 6.0) containing 1 mM NAD^+^ (Sigma-Aldrich, ref. N7004). rSasF was diluted to 20 μM in 1× dPBS (pH 7.0). The 20 μM rSasF protein (20 μl) was added to 180 μl of the above sugar-MBu substrate solution from above and allowed to incubate at 37°C for 3 hours for the α-GalNAc-MBu anomer experiments or used immediately for the kinetics experiments with the β-GalNAc-MBu anomer. Immediately, or after incubation at 37°C, the 100 μl of the reaction was transferred to a ultraviolet-transparent 96-well plate (Corning, ref. 3635). β-*N*-acetylglucosaminidase S (NEB, ref. P0744) or α-*N*-acetylgalactosaminidase (NEB, ref. P0734) was diluted 1:100 in 1× glycobuffer, and 2 μl of the 1:100 diluted enzyme was added to the sugar-MBu substrate solution. Immediately, the sample was inserted into a Tecan Infinite F200 plate reader, shaken linearly for 4 s at 3.5-mm amplitude, and read kinetically at every 5 s to track the enzyme reaction rate. The samples were read at a 375-nm excitation wavelength with a 445-nm emission wavelength with an excitation bandwidth of 9 nm and emission bandwidth of 20 nm. The gain was set to 70 with 10 flashes, a 20-μs integration time with a 20,000-μm *Z*-position. For the β-GalNAc-MBu MBu production kinetic experiments, a standard curve of free MBu was obtained. For the standard curve, a 1 mM stock of free MBu (TCI, ref. M0453) was prepared in the same buffer (also containing NAD^+^) as the above sugar substrates. This 1 mM stock was diluted serially 1:2 from 1000 to 0.49 μM, again in the same buffer. The concentration of MBu produced was calculated using the linear regression equation of the fluorescence signal over the concentration of free MBu from the standard curve (fig. S11). For the NADH addition experiments, the reactions and experiments were set up the same as above, except that NADH (Thermo Fisher Scientific, ref. AAJ6146103) was added to 5 mM instead of NAD^+^, a colorimetric β-GalNAc-pNA substrate was used at 1 mM (AmBeed, ref. A700641), and β-N acetylglucosaminidase S was diluted 1/200 instead of 1/100. The release of pNA was measured every 10 s on a Tecan Infinite F200 at OD_405_ with 10 flashes and a 9-nm bandwidth, and the no-enzyme control blank was subtracted from the experimental measurements to generate the plots in fig. S11. Data are displayed as the raw increase in fluorescence or absorbance measured over time.

### Isothermal titration calorimetry

To measure any interactions between rSasF and oleic acid, isothermal titration calorimetry was performed between rSasF and oleic acid using a Microcal ITC200 (Malvern Panalytical) device. The device was operated according to the manufacturer’s recommendations with rSasF at a concentration of 10 μM in the sample cell. A stock of either 100 μM or 3 mM oleic acid vortexed as much as possible in 1× DPBS (Corning, ref. 20-031-CV) was injected into the sample cell. The run using 100 μM oleic acid was stopped after 10 injections as this experiment showed heat evolution akin to that of water being injected into water. The 3 mM oleic acid experiment was conducted with 19 injections to generate a complete thermogram. The protein dilution in the sample cell and oleic acid stocks was used from a preserved aliquot of 1× DPBS from the final dialysis step when purifying rSasF to match buffer salt concentration between the DPBS in the sample cell and the DPBS in the injectant.

### rSasF unfolding and refolding

rSasF was purified as above and unfolded by resuspending concentrated protein in 8 M urea and 100 mM NaCl and heating the sample at 50°C for 10 min. Unfolded protein was separated from any bound NAD by running the sample over a Bio-Rad Enrich SEC70 10 × 300 column (Bio-Rad, ref. 7801070) equilibrated with degassed 8 mM urea and 100 mM NaCl. Before equilibrating with 8 mM urea and 100 mM NaCl, the column was equilibrated with degassed 100 mM NaCl, and column function was confirmed by running a gel filtration standard over the resin (Bio-Rad, ref. 1511901). After running the unfolded rSasF protein over the column, the protein collected and refolded by diafiltration using an Amicon Ultra-15 30K MWCO filter (Sigma-Aldrich, ref. UFC9030), spinning the sample down until it reached a 900 μl volume, and then resuspending the protein to 9 ml total in 1× DPBS. This process was repeated six times total to get rid of the urea and replace the sample buffer with 1× DPBS. The protein was then dialyzed two times at 4°C for 24 hours each against 2 liters of 1× DPBS using a Slide-a-lyzer 10,000 MWCO cassette (Thermo Fisher Scientific, ref. 66380). The final dialyzed protein was concentrated to 60 μM using a 30K MWCO filter as measured by BSA as above. The refolded and dialyzed protein was flash frozen as 20-μl beads and stored at −80°C until use.

### NADH fluorometric binding analysis

Refolded SasF was diluted to a working concentration of 50 nM in 1× DPBS. All measurements were performed in 1× DPBS. NADH (Thermo Fisher Scientific, ref. AAJ6146103) was dissolved in the same buffer to 1.5 × 10^−4^ M and kept on ice, protected from light. Steady-state fluorescence was recorded with a Fluorolog-2 spectrofluorometer (Horiba Jobin-Yvon) equipped with a Microlab 500-series automated titrator (*86*) fitted with a 250-μl stainless-steel Hamilton syringe. The protein sample (2.00 ml) was loaded into a 1-cm path-length quartz cuvette and thermostatted to 25°C with a Peltier controller while being constantly stirred. Six sequential injections of NADH stock (10, 10, 20, 50, 100, and 250 μl) were delivered at 65-s intervals. Excitation and emission wavelengths were fixed at 340 nm (2-nm band-pass) and 503 nm (10 nm band-pass), respectively, using a xenon arc lamp as the excitation source. Photomultiplier gain was set to 950 V, and no lamp-intensity correction was required because the lamp output remained stable throughout the experiment. Fluorescence intensity was integrated for 1 s and logged continuously for 65 s after each injection. Visual inspection showed that mixing artifacts subsided within ∼5 s. Therefore, the signal was averaged over 10 to 65 s for subsequent analysis. A blank trace obtained before any NADH addition (buffer only) was subtracted from all readings.

For the negative control and baseline correction, an identical NADH titration was performed in the absence of protein to quantify the intrinsic fluorescence of free NADH. The control fluorescence versus total NADH concentration was fitted to a first-order polynomial (MATLAB polyfit, order of 1). The fitted baseline was subtracted point by point from the protein trace to yield the binding-specific fluorescence change (Δ*F*). Inner-filter effects were not corrected because absorbance at 340 and 503 nm remained below 0.05 throughout the experiment. Δ*F* values were fitted to the quadratic 1:1 binding model $\Delta F_{i}=\Delta F_{\text{max}}\;\left[\frac{P_{0}+L_{i}+K_{d}-\sqrt{{\left(P_{0}+L_{i}+K_{d}\right)}^{2}-4P_{0}L_{i}}}{2P_{0}}\right]$, where *P*_0_ is the fixed protein concentration (50 nM) and *L*_i_ is the total NADH concentration after each injection. Nonlinear least-squares fitting was carried out in MATLAB R2023a (lsqcurvefit, trust-region-reflective algorithm) without weighting. Parameter confidence intervals (95%) were estimated with nlparci using the Jacobian returned by the fit.

### SasF amino acid conservation analysis

Amino acid conservation was calculated using ConSurf (*87*) using a protein alignment from the top 500 NCBI entries for a BLAST search against the FPR3757 SasF amino acid sequence. The protein sequence displayed is colored by conservation score at each amino acid position on the AlphaFold 3 SasF–predicted structure.

### *sasF* NaCl promoter activity and growth analyses

The AH1263 pCM29::P*sasF* reporter strain was grown in normal TSB overnight as above. The bacteria were then subcultured 1:100 in TSB with the indicated concentration (w/v) of NaCl. All TSB contains 40 mM Mops (pH 7.0) for GFP stability. Replicates (200 μl) of the diluted cultures were added to a black 96-well plate as above and grown at 37°C shaking at 800 rpm as above. Every hour the OD_600_ was measured using 10 flashes and a 9-nm bandwidth. To measure GFP production, the samples were excited at 492 nm with a 9-nm bandwidth and measured at an excitation wavelength of 539 nm with a 20-nm bandwidth. The gain was set at 60 with 10 flashes and a 20-μs integration time. Shown are the final OD_600_ and RFU (fluorescence/OD_600_) after 24 hours of growth with fluorescence signal serving as a proxy for the total *sasF* promoter activity throughout the entire experiment.

### Isolation of MRSA sacculi

Sacculi were first purified, as has been previously described (*88*) with minor modifications from equal volumes of overnight cultures of cells grown in either normal TSB or TSB and NaCl (75 g/liter; ∼1.2 M). Briefly, 5-ml cultures of MRSA were spun down and resuspended in 5 ml of 150 mM tris (pH 7.4) and 2% (w/v) SDS. The samples were boiled for 10 min and allowed to cool then spun down. The pellets were resuspended in 3 ml of sterile Milli-Q H_2_O. Each sample (1.5 ml) was aliquoted to 2-ml bead-beater tubes containing 0.1 mM silica beads and homogenized four times in 1-min intervals with cooling on ice in between each interval. The bead-beat samples were centrifuged at 100*g* for 2 min, and the supernatant was removed and pooled for each sample. The samples were then spun down and washed twice with 150 mM tris (pH 7.4) and 2% SDS and then another six times with sterile Milli-Q. The washed samples were then resuspended in 1 ml of 100 mM MES (pH 6.5) and 150 mM NaCl.

### MGL staining of purified MRSA sacculi

Purified saculi (100 μl) were applied to Ibidi μ-slide 18 well ibiTreat chamber slides (Ibidi, ref. 81816) and allowed to incubate at RT for 1 hour. The samples were aspirated, and the 100 μl of blocking solution [1× TBS, 1% (v/v) Tween 20, and 5% BSA] was added to each well and allowed to incubate for an hour at RT. The blocking solution was aspirated, and hMGL (5 μg/ml; R&D Systems, ref. 4888-CL) in 10 mM tris (pH 7.2), 100 mM NaCl, and 2 mM CaCl_2_ (or buffer only for the secondary-only staining controls). The samples were incubated at RT for 1 hour and then aspirated. Each well was washed twice with 10 mM tris (pH 7.2), 100 mM NaCl, and 2 mM CaCl_2_, and, then, an anti-His Alexa Flour 647 monoclonal secondary antibody (Thermo Fisher Scientific, ref. MA1135A647) was added to each well at 1/800 in 10 mM tris (pH 7.2), 100 mM NaCl, and 2 mM CaCl_2_, allowed to incubate for 1 hour at RT, and then aspirated. Each well was washed twice with 10 mM tris (pH 7.2), 100 mM NaCl, and 2 mM CaCl_2_ and then imaged by microscopy immediately at 20×. All images were analyzed using ImageJ. Each image for the fluorescence channel was set to 8 bit and then set to the Otsu threshold. The % stained area was calculated in ImageJ and was used for the analyses herein.

### Skin abscess formation model of skin infection

One day before infection, the stomachs of 7-week-old female BALB/C mice were shaved, and a fingertip amount of Nair was applied evenly to the shaved area for depilation. After 30 s, a fresh paper towel was used to remove the residual Nair from the animal’s stomachs. The animals were then allowed to rest overnight. On the day of infection overnights of the bacterial strains of interest were subcultured 1:100 in 20 ml of sterile TSB and allowed to grow at 37°C shaking 220 rpm to mid-log (OD_600_ of 0.4 to 0.6). The mid-log cultures (15 ml) were spun at 4000*g* for 10 min at 4°C. The supernatant was removed, and the pellet was washed in 10 ml of cold PBS. The cultures were then spun down and resuspended twice more for a total of three washes. The cultures were then adjusted to ∼1 × 10^8^ CFU/ml in cold PBS. The skin of each shaved mice was briefly wiped with a 70% isopropanol wipe before injection and allowed to dry. Using a new sterile 33-gauge syringe for each animal, 50 μl of the bacterial culture (1 × 10^8^ CFU/ml) was injected into each mouse intradermally. The mice were weighted and placed back into their cages, and the inoculum was serially diluted and plated for CFU on TSA. The plates were allowed to incubate inverted overnight at 37°C before counting bacterial colonies. For days 2 to 6, the abscess sizes were imaged next to a ruler and quantified, and the mouse weights were measured. At day 7, the abscesses were imaged, and the weights were measured before euthanizing the mice using CO_2_ asphyxiation followed by cervical dislocation. The skin over the abscess was sprayed with 70% ethanol and allowed to dry, and a 6-mm biopsy punch was used to remove the abscess from the mouse. The excised abscess was placed into a sterile, preweighed bead beating tube containing 500 μl of 1× PBS and ∼^1^/_4_-inch (0.635-cm)–thick layer of 1-mm zirconium beads (RPI, ref. 9835). The abscesses were bead beat for 1 min, placed on ice for 5 min, and then bead beat again for another min. The resulting slurry was serially diluted and plated on mannitol salt agar containing cefoxitin (2.5 μg/ml) as above to select for MRSA cells. The plates were inverted and incubated at 37°C overnight for CFU enumeration.

### Red blood cell hemolysis assay

An overnight culture of the indicated strains (5 ml) was spun down, and the supernatant was collected and filtered through a 0.22-μm filter. Defibrinated rabbit blood was purchased from HemoStat laboratories (ref. DRB100). To make the RBC solution, defibrinated rabbit blood and 1.3× DPBS (Corning, ref. 20-031-CV) were combined at a ratio of 1:5 blood:1.3× DPBS and mixed gently. The mixture was then centrifuged at 1000*g* at 4°C until the cells were pelleted. The supernatant was decanted, and the blood cells were washed as above until the supernatant was clear. After washing, the resulting pellet was considered a 100% red blood cell suspension. The red blood cells were diluted further to a 3% final volume of red blood cells in 1.3× DPBS, which was the working solution of red blood cells used. The filtered bacterial supernatants from above were then diluted twofold in a 30 μl total volume of 1.3× DPBS from 30% spent supernatant to 0.015% spent supernatant final supernatant concentrations. To all wells, 70 μl of the 3% red blood cell solution from above were gently added to the 30 μl dilutions of spent supernatants, and the plates were mixed gently by swirling the plate on a benchtop. The plate was allowed to incubate for 1.5 hours at room temperature, at which point the OD_630_ was measured using a Tecan infinite plate reader. From the data obtained, a four-parameter logistic fit was applied to each sample. For the agar plate-based hemolysis assay, a sterile 6-mm biopsy punch was used to create the wells in a TSA agar plate containing 5% sheep blood (BD, ref. 221261). Then, 50 μl of 0.22-μm-filtered bacterial supernatant was added to each respective well. The plate was then incubated overnight at 37°C.

### Structural modeling and comparison between MRSA SasF and *S. epidermidis* SasF

For structural modeling of MRSA SasF, the full protein sequences from *S. aureus* FPR3757 (GenBank, ABD21199.1) or *S. epidermidis* (NCBI, ref. WP_381410357.1) were modeled using AlphaFold 3 (*25*). For structural comparisons, both modeled protein structures were aligned one-to-one using the alignment plugin in The PyMOL Molecular Graphics System (version 3.1.5.1, Schrödinger LLC) with 5 cycles and a cutoff of 2.0, and the resulting alignment RMSD is reported above.

### Statistical analyses and photographic imaging

All statistics were performed using GraphPad Prism (version 10.1.1). The statistical tests used for piece of data are described in their respective figure legends throughout this manuscript. For significance, a *P* value of less than or equal to 0.05 was considered significant. Throughout the manuscript, * indicates *P* ≦ 0.05, ** indicates *P* ≦ 0.01, *** indicates *P* ≦ 0.001, **** indicates *P* ≦ 0.0001, and n.s. indicates no significance as determined by the indicated statistical test. The images for the skin abscess formation model were taken using a Canon Powershot ELPH180. All other images were taken using the cameras of an iPhone 14 Pro or iPhone 17 Pro Max (Apple Inc., California, USA). Microscopy was performed on a Keyence BZX710 fluorescent microscope.

### Animal ethics and supplier information

All mouse experiments were performed in the University of Colorado Anschutz Medical Campus’ ABSL2+ facility, with experiments approved under IACUC protocol no. 00941 for skin colonization experiments and no. 00486 for skin abscess formation model experiments. All mice were ordered from the Jackson Laboratory (Bar Harbor Maine, USA). All animals were euthanized humanely by CO_2_ asphyxiation followed by a secondary cervical dislocation as is recommended by the American Veterinary Medical Association Guide for the Euthanasia of Animals.

## Supplementary Materials

### This PDF file includes:

Figs. S1 to S16 Tables S1 and S2 References
