# Supplementary material for: Staphylococcus aureus wall teichoic acid structure dictates virulence potential and resistance to host defenses
Source: Sci Adv. 2026 Oct 21;12(43):eaef7070. doi: 10.1126/sciadv.aef7070 (PMC13685921; doi:10.1126/sciadv.aef7070)
Supplement: Supplementary file 1 [file sciadv.aef7070_sm.pdf]

Science Advances
AAAS

Supplementary Materials for

Staphylococcus aureus wall teichoic acid structure dictates virulence potential and resistance to host defenses

Timothy J. Enroth *et al.*

Corresponding author: Alexander R. Horswill, alexander.horswill@cuanschutz.edu

Sci. Adv. **12**, eaef7070 (2026)
DOI: 10.1126/sciadv.aef7070

This PDF file includes:

Figs. S1 to S16
Tables S1 and S2
References

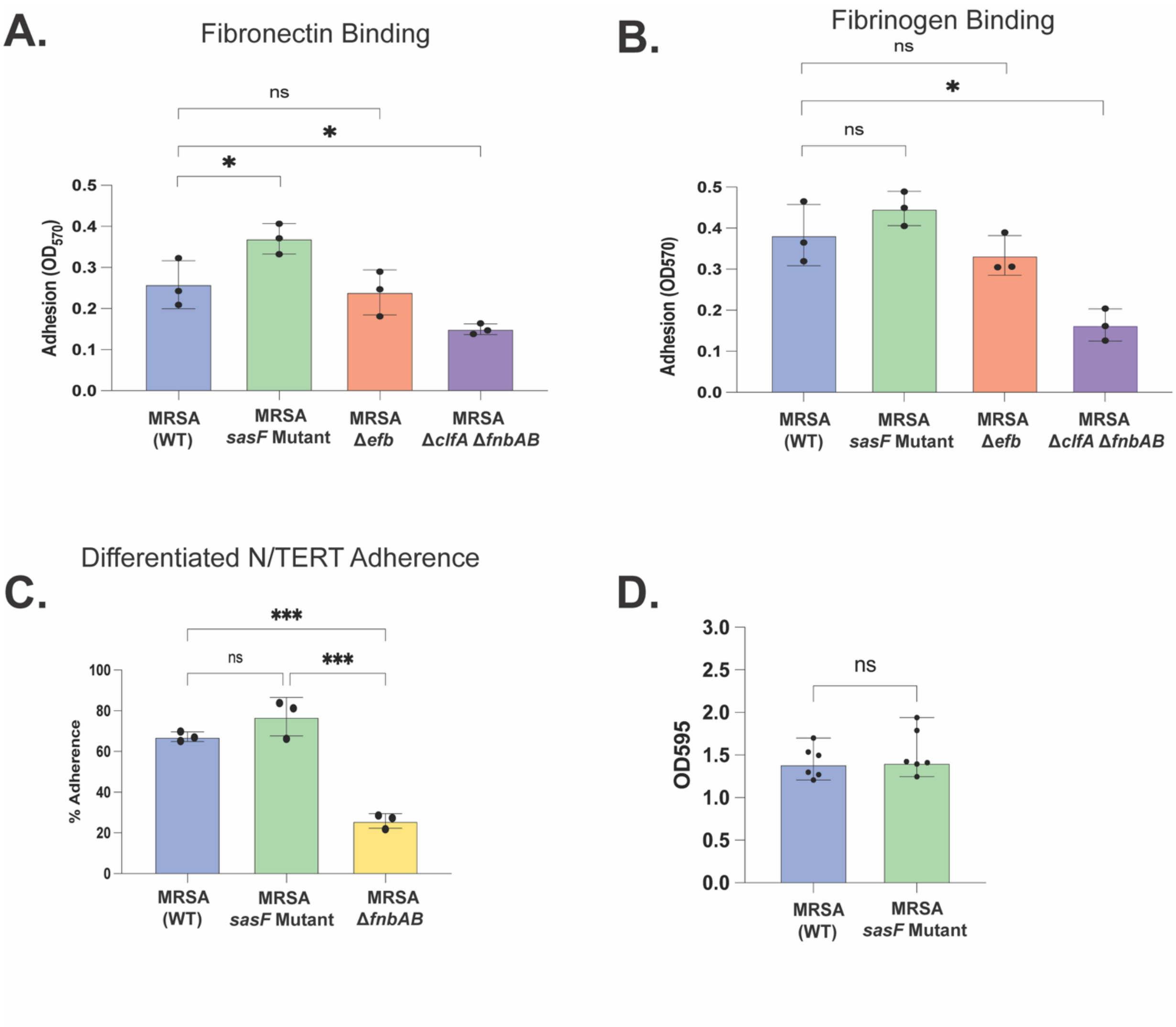

Figure S1: SasF does not behave as an adhesin in the models tested herein. A) Fibronectin binding assay and fibrinogen binding assay **(B)** results suggest that SasF does not bind these host matrix components. **C)** The MRSA *sasF* mutant is not less adhesive to differentiated N/TERT-2G cells differentiated at the air liquid interface using transwells compared to MRSA (WT). **D)** Crystal violet staining of bacterial biomass/biofilm is not significantly different for the MRSA *sasF* mutant compared to MRSA (WT), suggesting that SasF does not serve as a crucial biofilm matrix component. Shown are mean ±SD for three independent biological replicates and statistics were performed via ordinary one-way ANOVA for panels A-C, and a student's T-test for panel D.

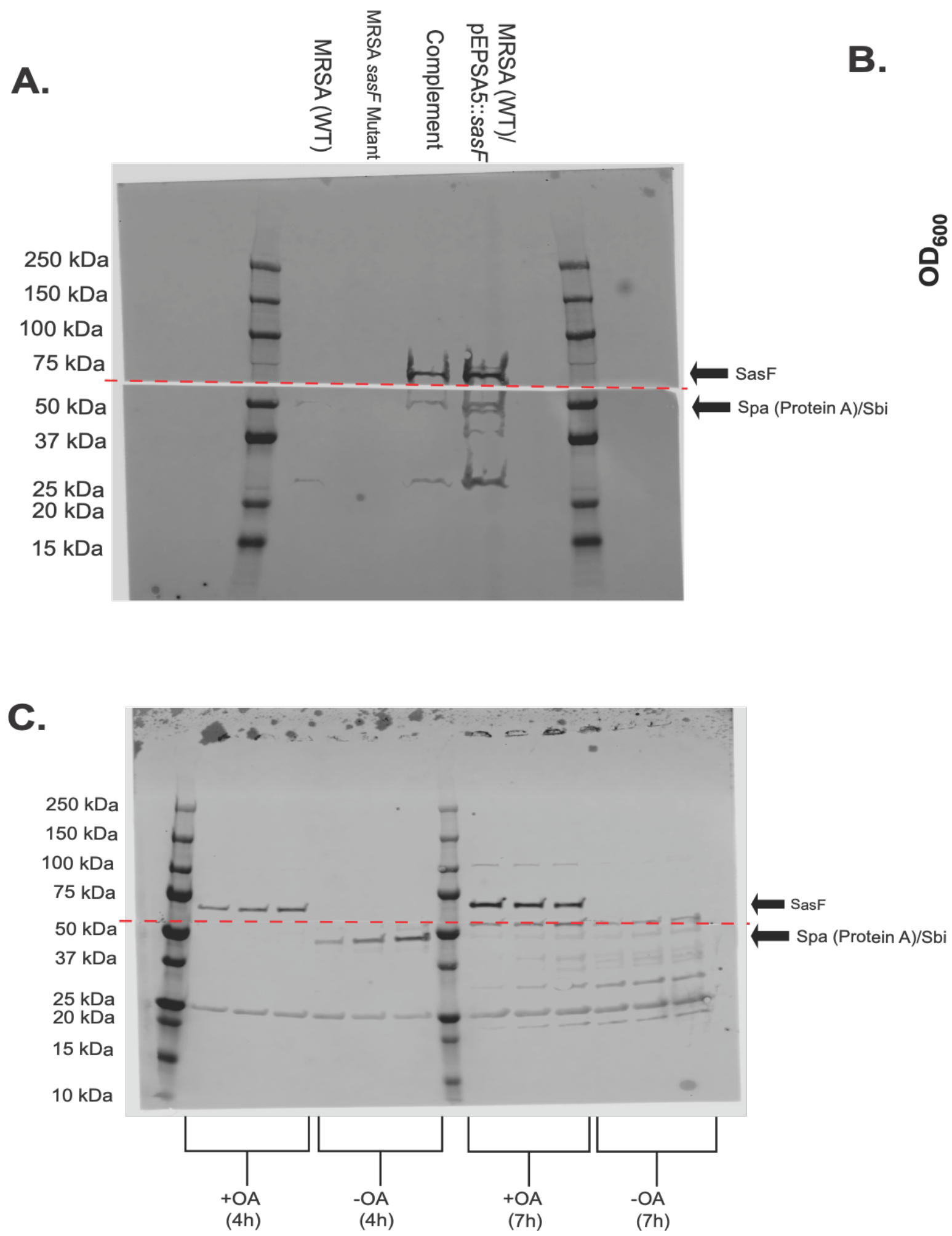

Figure S2: Confirmation of anti-SasF polyclonal antibodies and the post-transcriptional production of SasF in response to oleic acid. A) Western blot showing MRSA (WT), the *sasF* mutant, complement and a MRSA (WT)/ pEPSA5::*sasF* overexpression strain, confirming polyclonal antibody binding. **B)** Growth curve that accompanies Figure 1C*,* which was used to generate the promoter activity data. **C)** Full, uncropped Western blot from Figure 1C showing increased SasF protein production upon exposure to oleic acid. One biological replicate is shown for the polyclonal antibody confirmation in Figure S2A. Mean ±SD for three biological replicates shown for Figure S2B, and three biological replicates are shown for each condition on the same membrane for Figure S2C. The Western blots have been cut between approximately 50 and 60 kDa (red dashed line) to ensure that binding of antibodies by Spa and Sbi do not affect staining results for SasF, and the bands corresponding to Spa and Sbi are labelled.

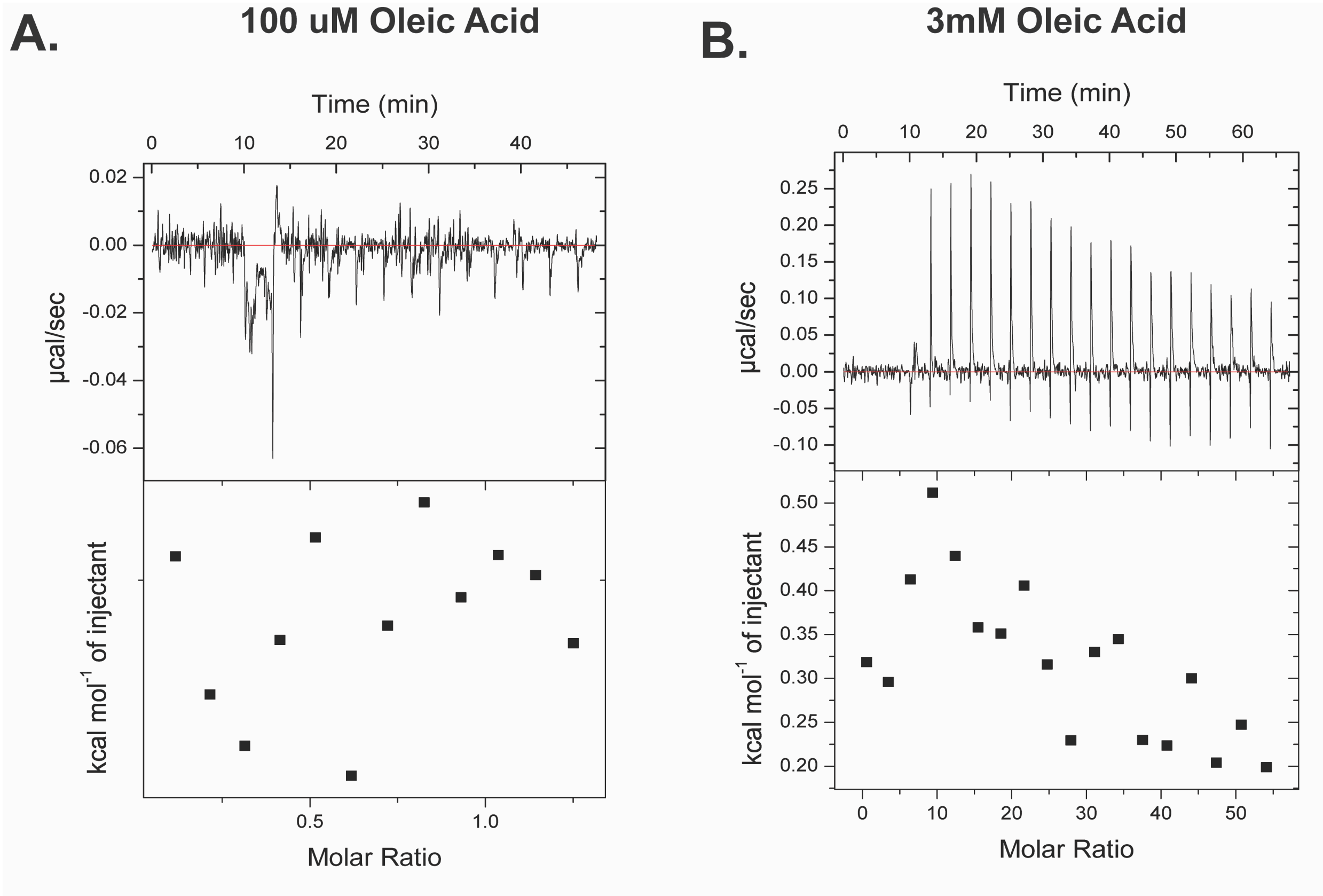

Figure S3: Isothermal titration calorimetry shows no direct interaction between rSasF and oleic acid. A) Thermogram of a 100 µM stock and 1 mM (B) of oleic acid in ethanol injected into a cell containing rSasF show an endothermic process resulting from oleic acid being introduced into the solvent, but not oleic acid interacting with rSasF. Shown are individual replicates using fresh protein and oleic stocks for each thermogram.

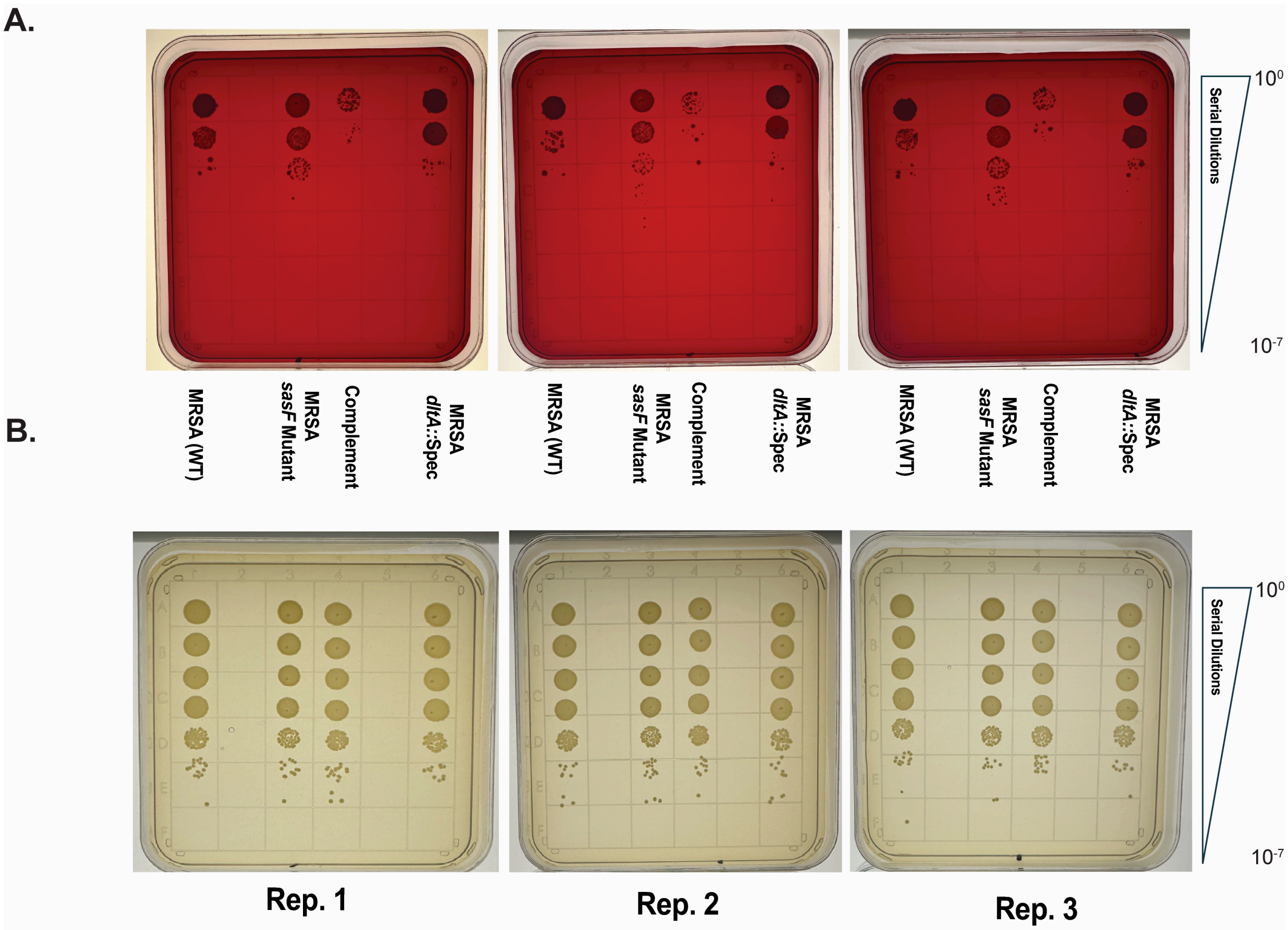

Figure S4: Overexpression of SasF results in increased Congo red binding to the bacterial cells. A) A MRSA *sasF* mutant is more resistant to 0.18% Congo red compared to MRSA (WT), and the complement strain is more susceptible to Congo red, despite all bacterial strains normalized to the same starting CFU/mL as indicated by equivalent CFU/mL when grown on TSA only **(B).** Shown are three independent biological replicates for each strain.

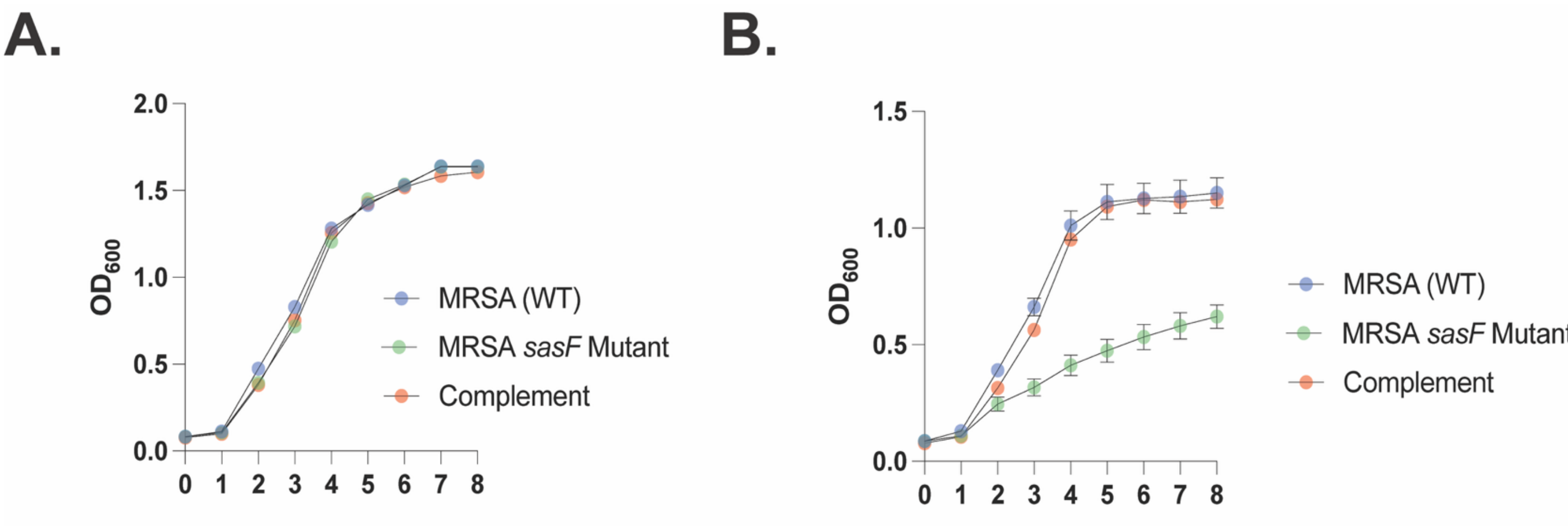

Figure S5: A MRSA *sasF* mutant has an impaired growth curve when grown in the presence of tunicamycin. A) TSB + DMSO solvent control growth curve of MRSA (WT), the MRSA *sasF* mutant and the complement strain show no difference in bacterial growth over time. **B)** When the MRSA *sasF* mutant is grown in the presence of tunicamycin the growth is significantly impaired compared to MRSA (WT), and this phenotype is complementable. Shown are mean ±SD for three independent biological replicates.

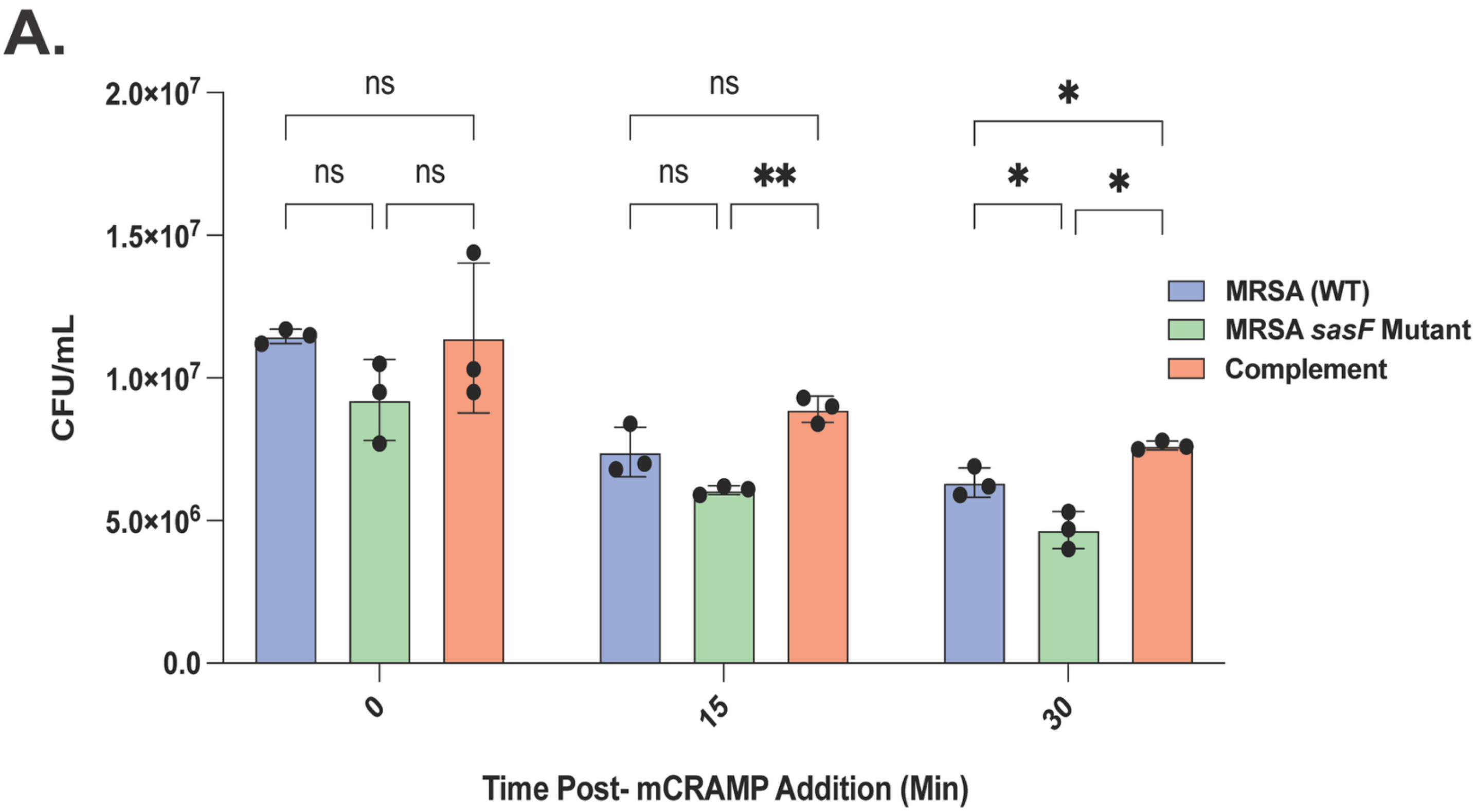

Figure S6: The MRSA *sasF* mutant is slightly more sensitive to the amphipathic antimicrobial peptide, mCRAMP. A) Analysis of CFU/mL over time of the MRSA *sasF* mutant incubated with 40 µM mCRAMP shows that MRSA *sasF* mutant is modestly more sensitive to the antimicrobial peptide compared to MRSA (WT), and that this phenotype is complementable. Shown are mean ± SD. Statistics are measured by a two-way RM-ANOVA. Shown are the results from three independent biological replicates.

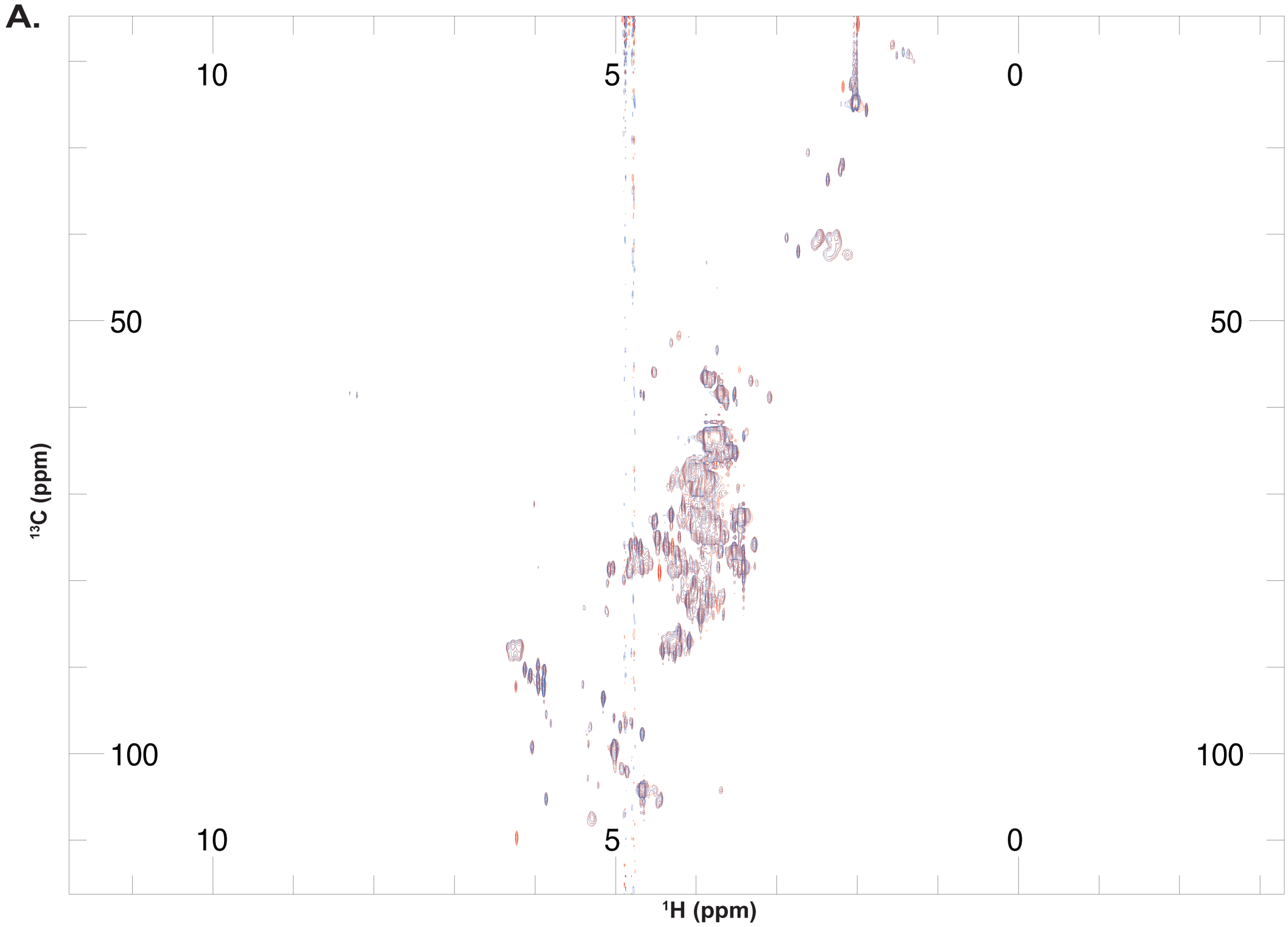

Figure S7: Full HSQC spectrum of WTA from the *sasF* mutant with and without rSasF addition. A) Full HSQC spectrum of ^{13}C-enriched WTA from the *sasF* mutant that were obtained pre- (red) and post- (blue) addition of rSasF. Highlighted are chemical shifts that can be reasonably assigned given previous NMR experimental data**.**

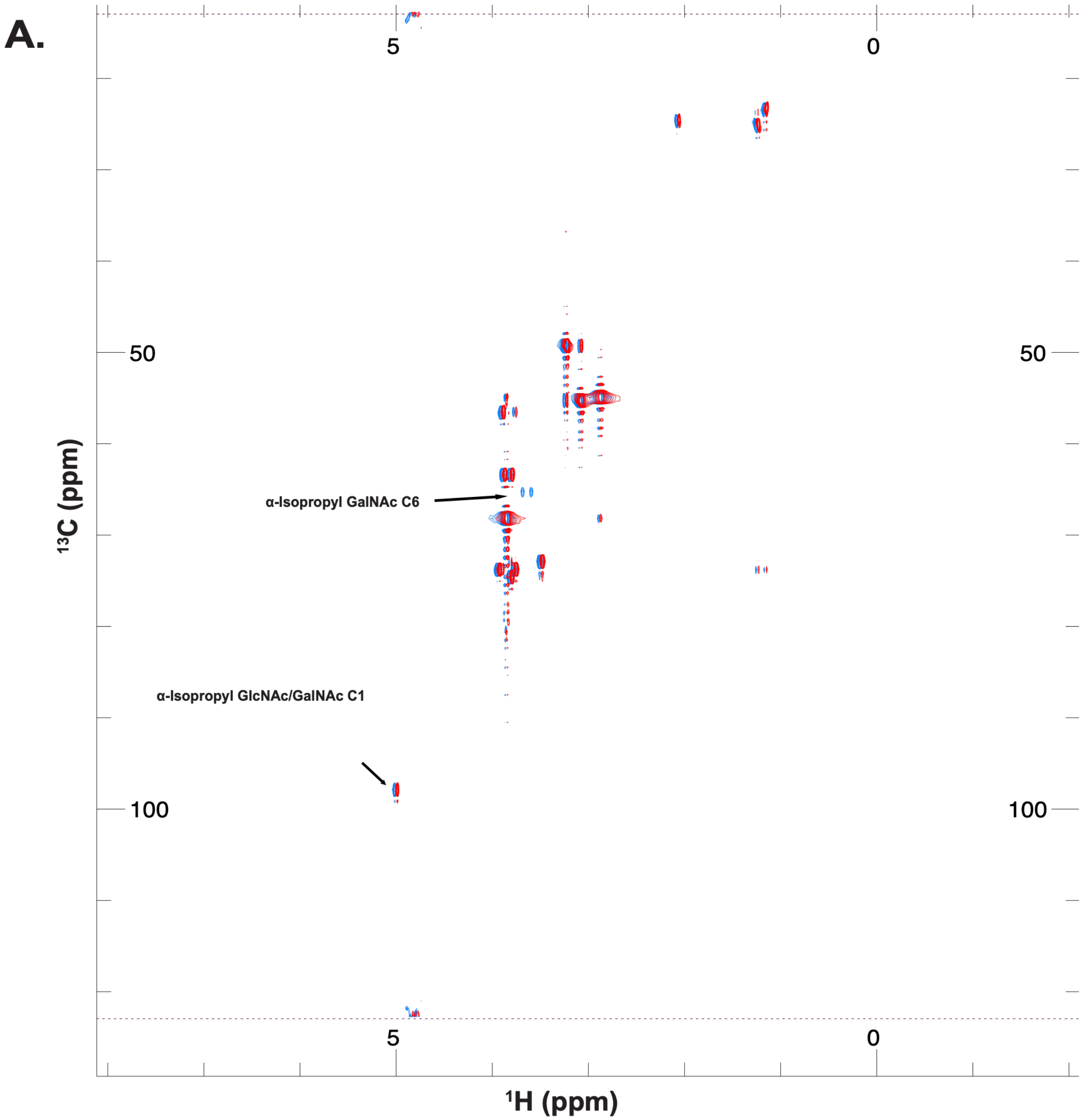

Figure S8: Full HSQC spectrum of α-isopropyl GlcNAc with and without rSasF addition.
A) Full HSQC spectrum of the α-isopropyl GlcNAc anomer that were obtained pre- (red) and post- (blue) addition of rSasF using naturally-abundant ^{13}C.

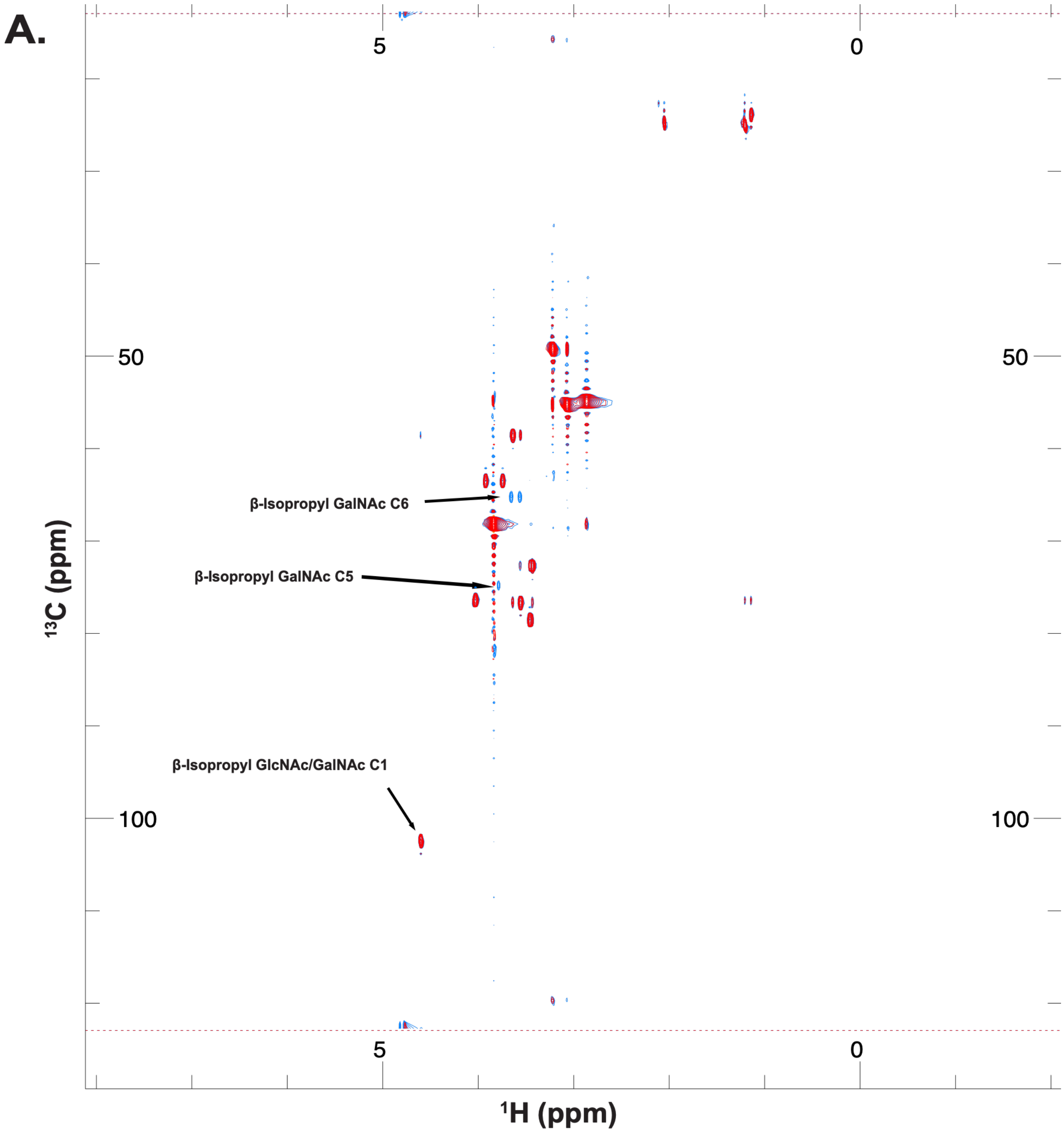

Figure S9: Full HSQC spectrum of β-isopropyl GlcNAc with and without rSasF addition. **A)** Full HSQC spectrum of the β-isopropyl GlcNAc anomer that were obtained pre- (red) and post- (blue) addition of rSasF using naturally-abundant ^{13}C.

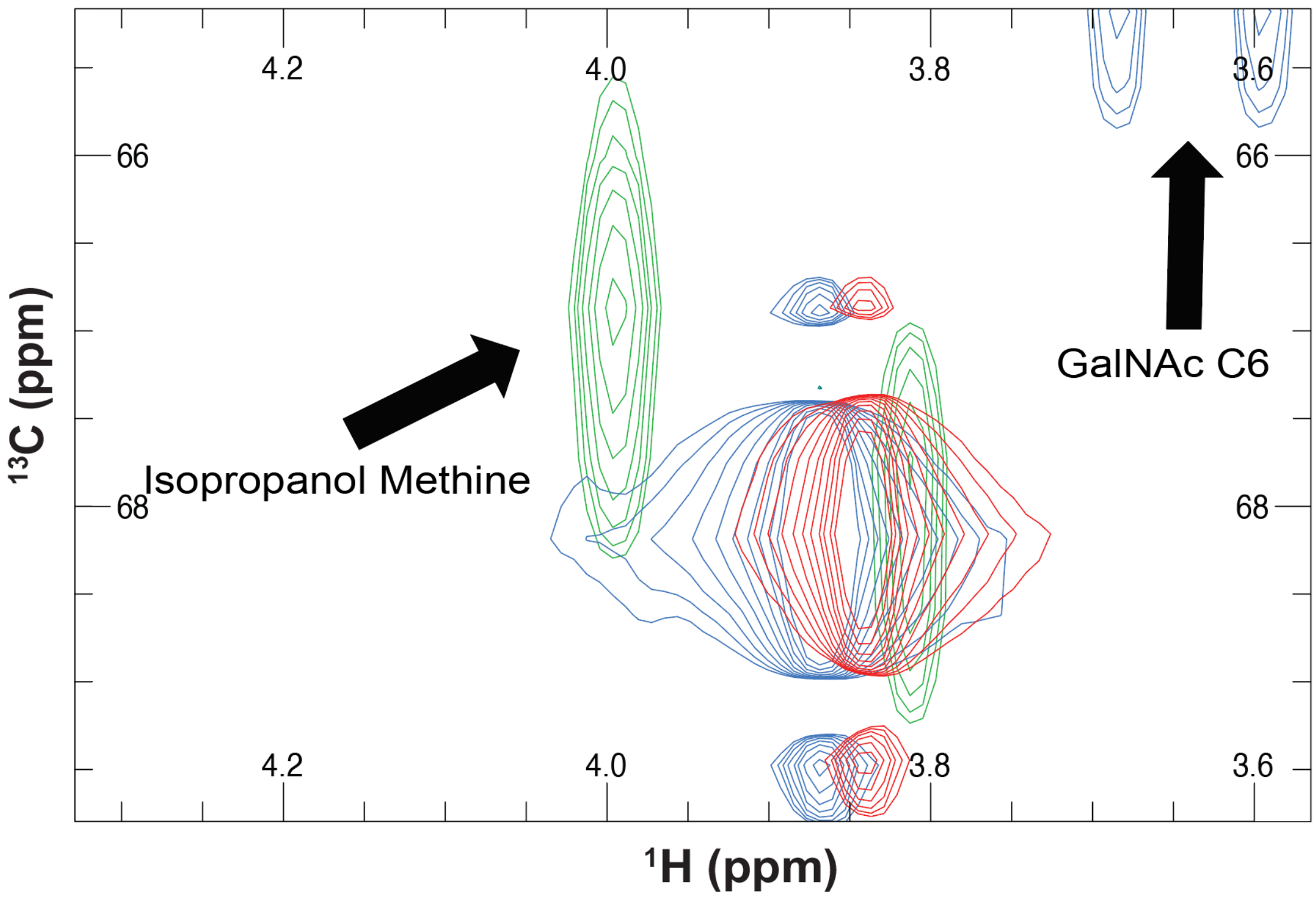

Figure S10: Comparison of α-isopropyl GlcNAc spectrum to an isopropanol control shows no cleavage of isopropanol from α-isopropyl GlcNAc upon rSasF addition. A) HSQC spectrum of the α-isopropyl GlcNAc anomer that were obtained pre- (red) and post- (blue) addition of rSasF compared to the methine chemical shift of free isopropanol. There is no overlap between the isopropanol methine chemical shift, the GalNAc C6 diastereotopic protons, and GalNAc C4 that appear upon rSasF treatment, suggesting that rSasF addition to isopropyl GlcNAc substrates does not result in glycosidic cleavage at C1.

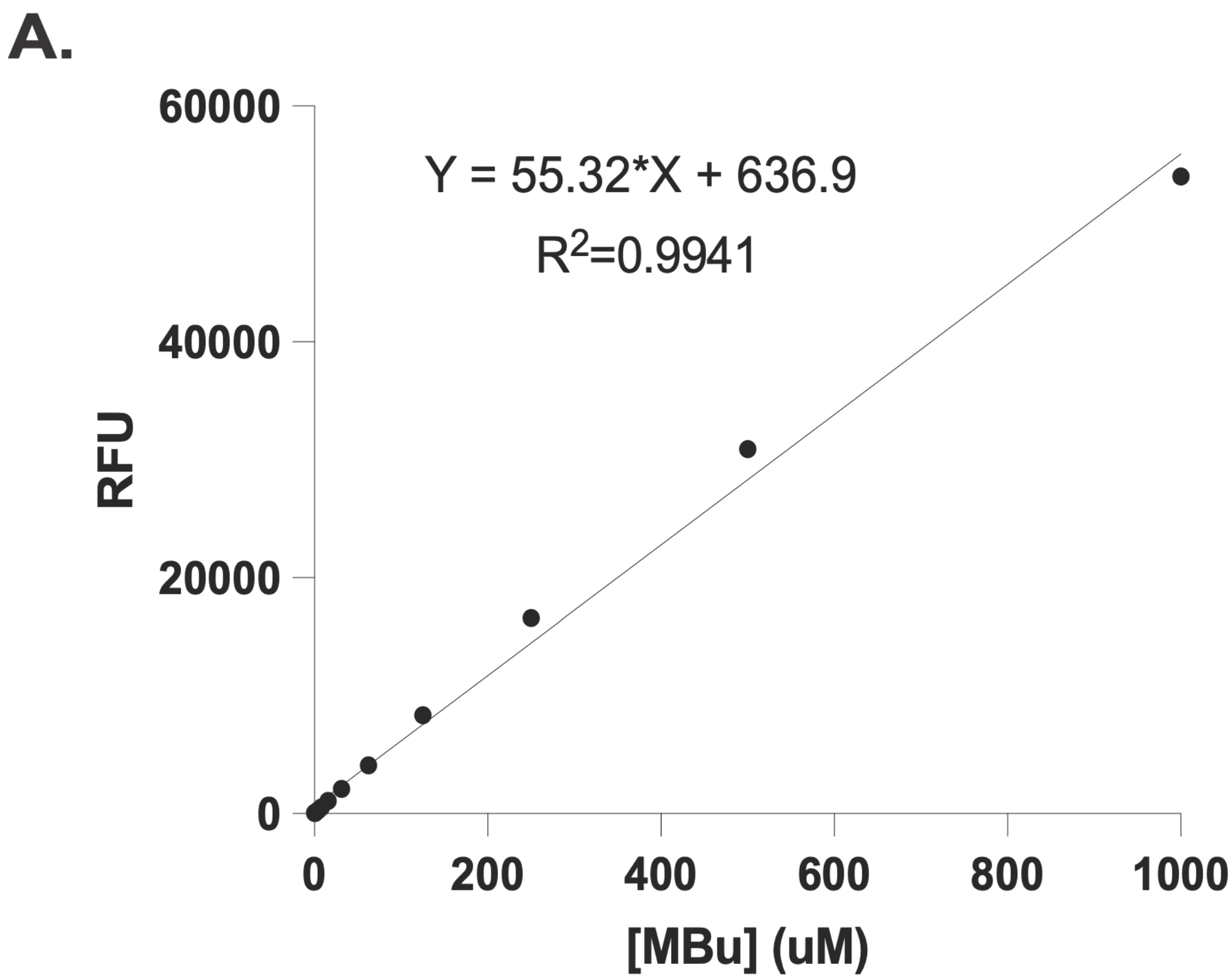

Figure S11: Standard curve of free 4-methylumbelliferone. A) A standard curve of a serially diluted 1 mM stock of free fluorescent 4-methylumbelliferone was created in the same buffer conditions and measured at the same wavelengths as in **Figure 3E**. Shown are mean ±SD for three independent replicates. Error bars are hidden behind the symbol.

A.

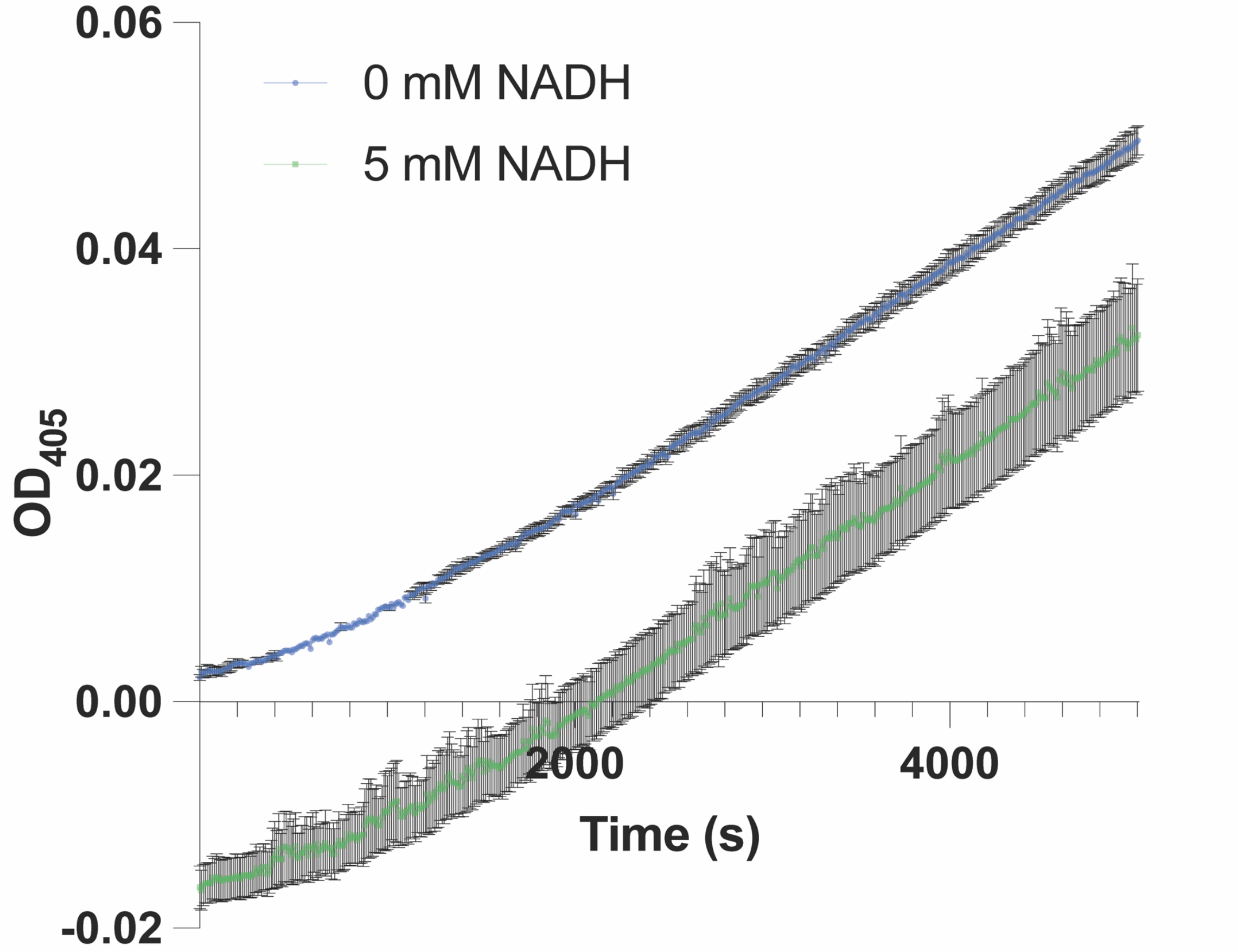

Figure S12: MRSA SasF Enzyme Activity is Poisoned by NADH. A) When excess NADH is added to GalNAc-pNA, rSasF isomerization activity is significantly impaired, with detectable signal only observable at approximately 30 minutes post-β-N-Acetylglucosaminidase S addition. Shown are mean ±SEM for three independent replicates, and the data shown are the OD_{405} values with a no-rSasF control blank subtracted.

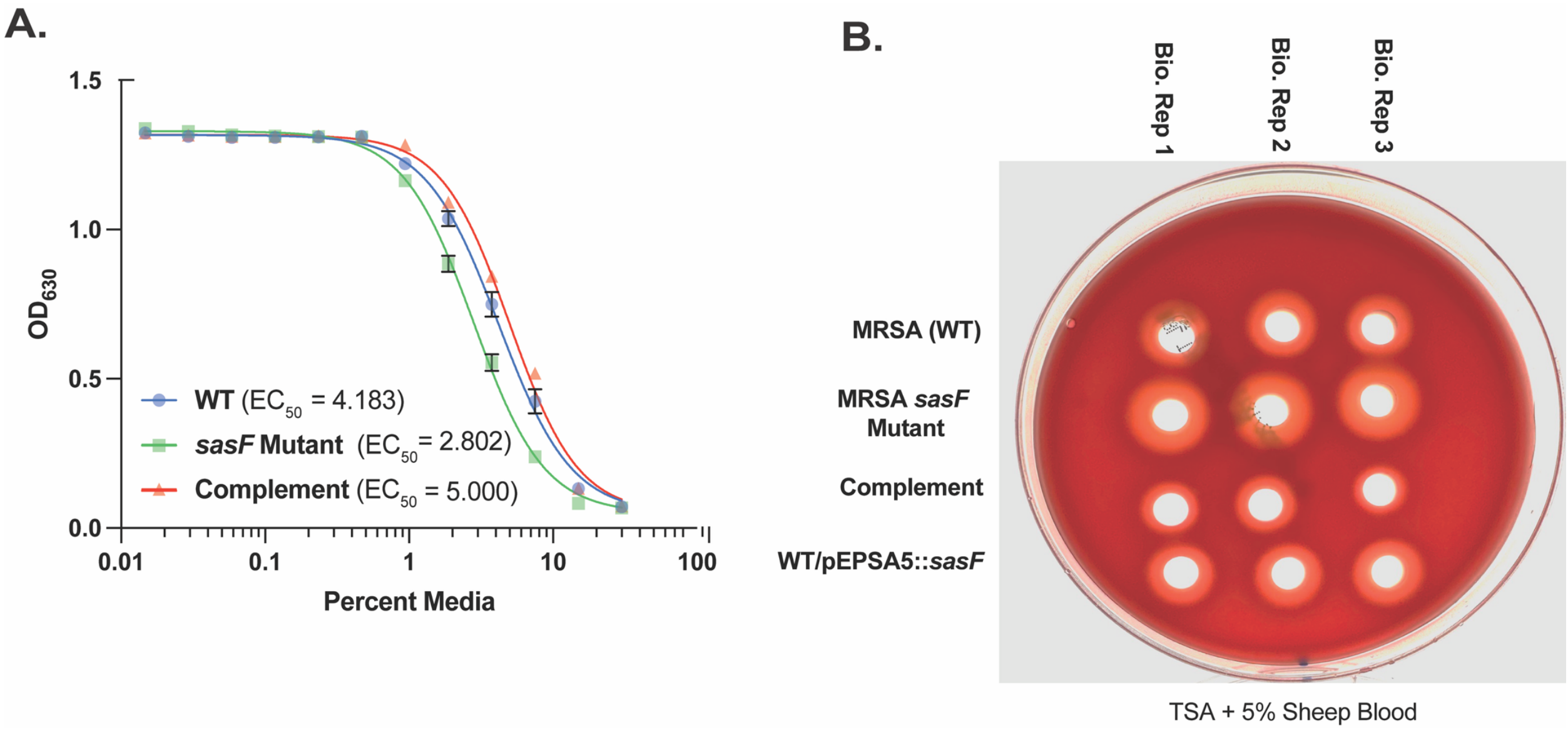

Figure S13: A MRSA *sasF* mutant is slightly more hemolytic compared to MRSA (WT). A) Blood cell lysis curves with a four-parameter logistic fit suggests that the MRSA *sasF* mutants supernatant is approximately 1.5-fold more hemolytic to defibrinated rabbit blood compared to MRSA (WT). **B)** Incubation of 50 uL of supernatant from the MRSA *sasF* mutant in a 6 mM diameter well punched in a blood agar plate support the findings in panel A, showing more hemolysis caused by the MRSA *sasF* mutant supernatant. Each panel show the results from three independent biological replicates. The mean ±SEM are shown in panel A.

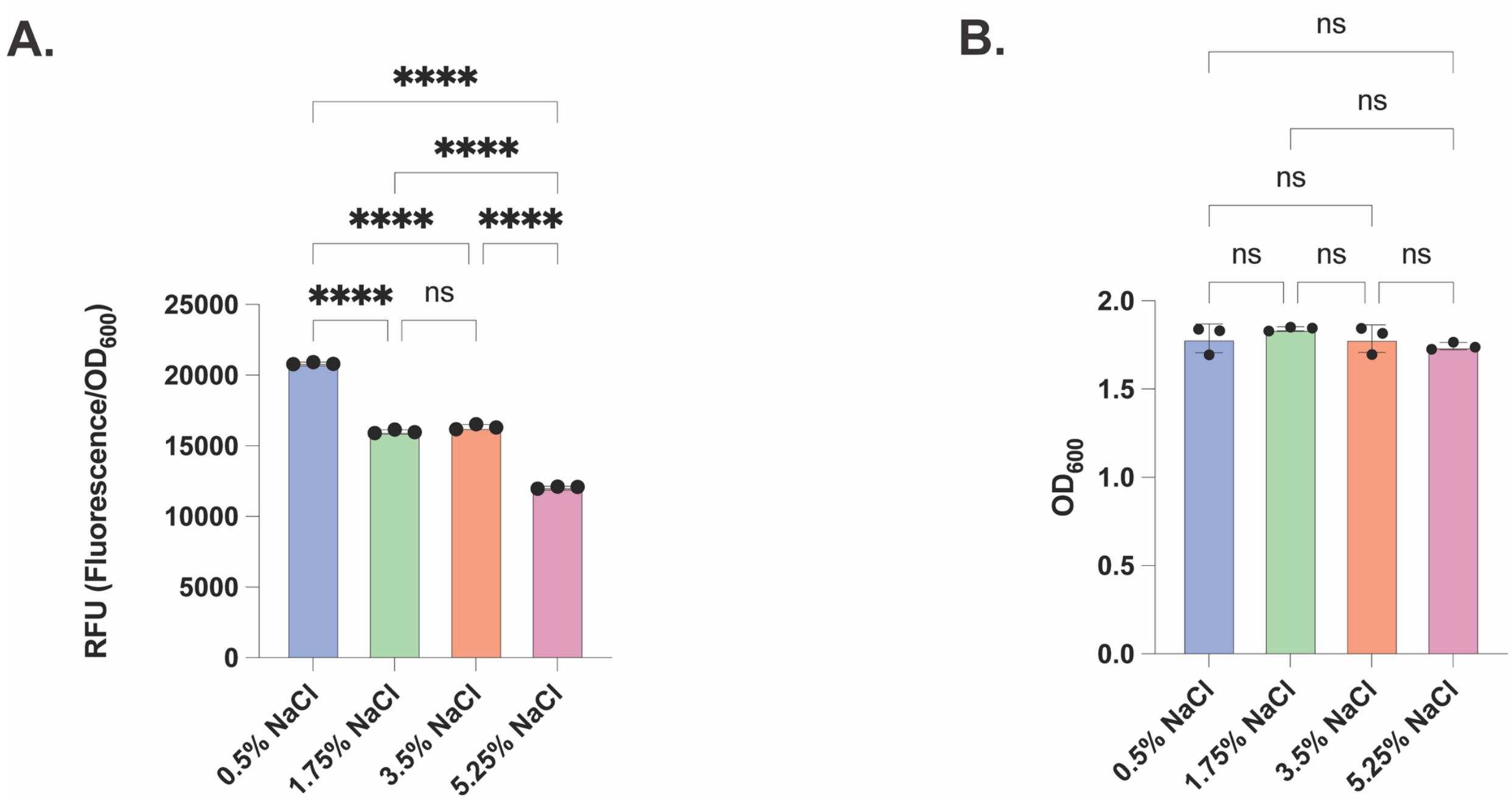

Figure S14: *sasF* Promoter Activity is Decreased Under High Salinity Whilst Growth Remains Unchanged. A) *sasF* promoter activity analysis at 24 hours suggests that when cells are grown in high NaCl concentrations, *sasF* promoter activity decreases significantly. **B)** Growth of *S. aureus* in increasing NaCl concentrations is not different at 24 hours, suggesting that promoter activity differences in A are not due to differences in bacterial abundance. Shown are mean ±SD for three independent biological replicates. Statistical analyses were performed via an ordinary one-way ANOVA for both panels.

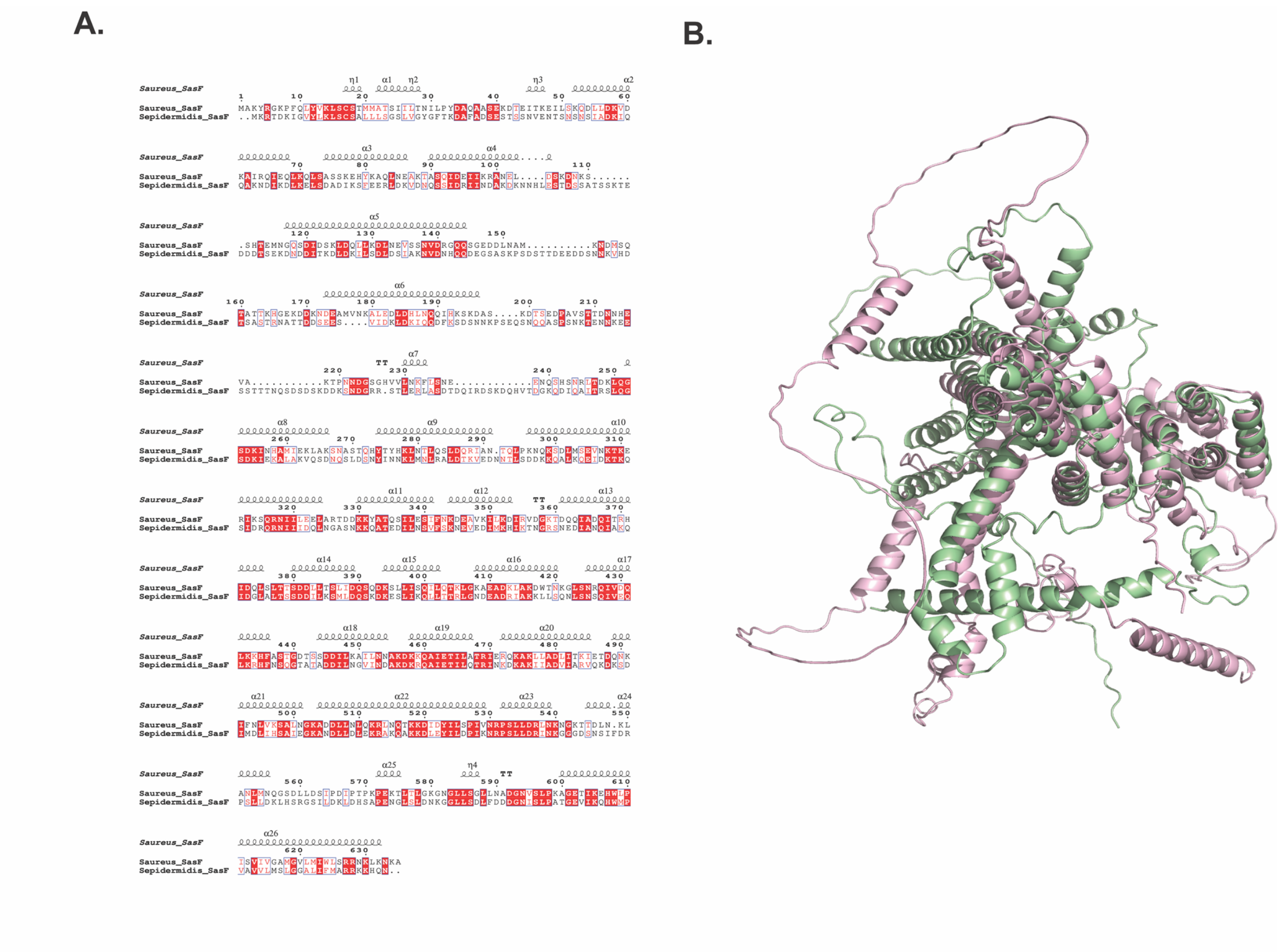

Figure S15: SasF from MRSA and *S. epidermidis* are Structurally Distinct. A) Amino acid Clustal Omega sequence alignment between MRSA LAC SasF and a SasF homologue from *S. epidermidis.* **B)** Superposition of AlphaFold 3 predicted structures of MRSA LAC SasF (green) and a SasF homologue from *S. epidermidis* (pink) (RMSD=4.315).

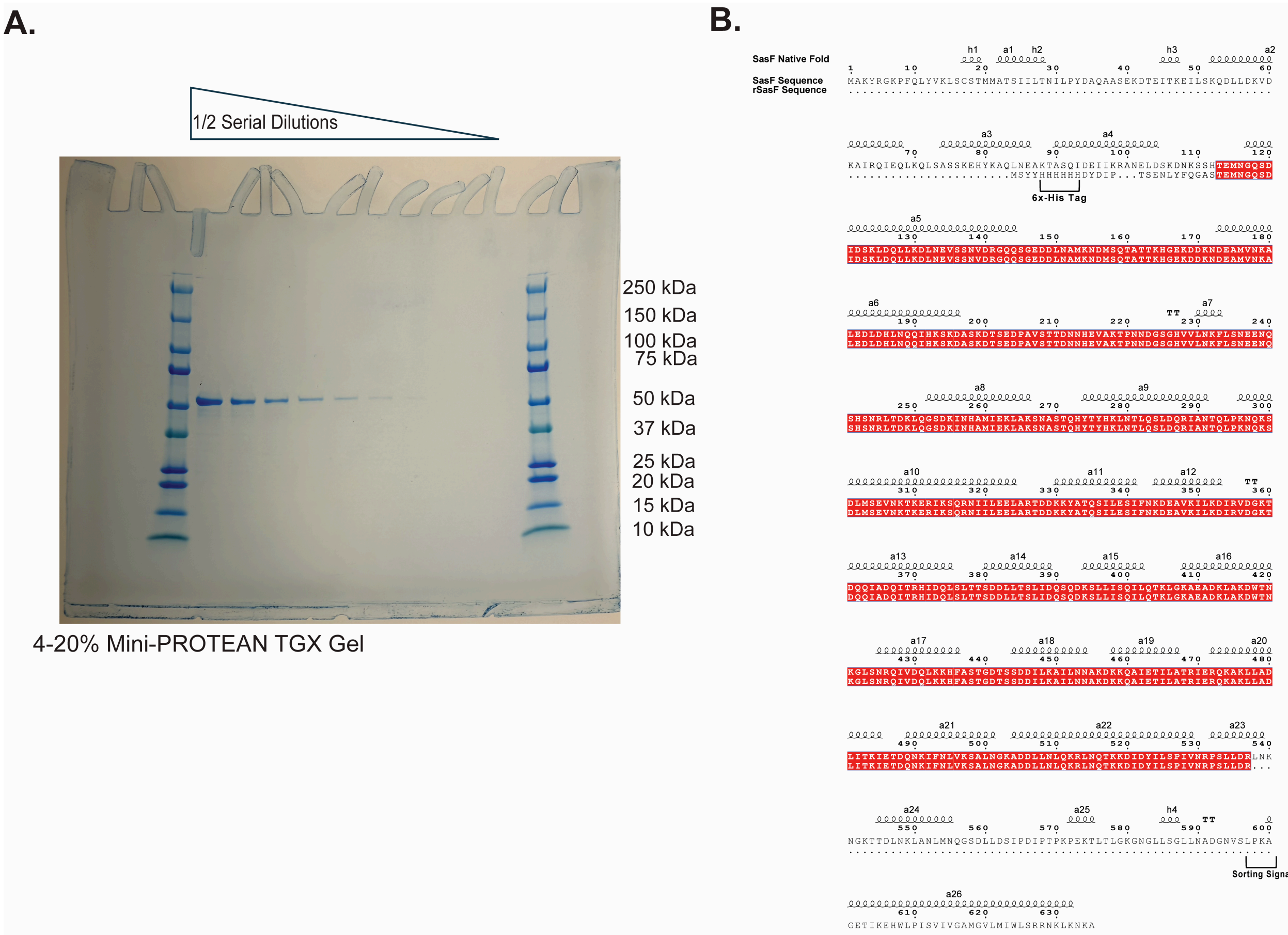

Figure S16: SasF Purity and Sequence as Purified. A) Coomassie-stained 4-20% SDS-PAGE gel of a rSasF dilution series from 508 µM to 248 pM, with 12 uL of each dilution added per lane and compared to a BioRad Precision Plus Protein Kaleidoscope Ladder (BioRad ref: 1610375). **B**) Protein sequence comparison between native SasF and rSasF, aligned using Clustal Omega(89) and displayed using ESPript 3.0(90). The amino acids that comprise purified rSasF are highlighted in red.

Table S1: List of Strains and Plasmids Generated and Used

Strain and Plasmid Names	Description	Source
Escherichia coli<br>NEB 5-alpha	*recA*-deficient *E. coli* cloning strain	NEB
AH3254	*E. coli DH5*α containing pTEV5 (EV)	Lab Stock
AH0003	*E. coli* ER2566	Lab Stock
AH0120	*E. coli* BL21 (DE3) STAR	Lab Stock
AH4532	*E. coli* DC10B containing pCM28 (EV)	Lab Stock
TJE215	*E. coli* DH5α/pTEV5::$sasF^{\Delta N\Delta C}$	This Study
TJE441	*E. coli* ER2566/pTEV5::$sasF^{\Delta N\Delta C}$	This Study
TJE71	*E. coli* BL21 (DE3) STAR/pTEV5::$sasF^{\Delta N}$	This Study
AH5027	*E. coli* DC10B containing pCM29	(91)
Staphylococcus aureus<br>AH1263	MRSA (WT)	(92)
AH5996	MRSA *sasF::*Tn (MRSA *sasF* mutant)	This Study
AH1292	MRSA *agr::*tet	(93)
AH4393	LAC Δ*clfA*Δ*fnbAB*	(94)
AH4392	LAC Δ*fnb*AB	(94)
AH4397	LAC Δ*efb*	Heidi Crosby
AH4398	LAC Δ*clfA* Δ*efb*	Heidi Crosby
AH2235	SA112 *dltA*::Spec	Peschel lab
TJE102	MRSA *sasF::*Tn/pCM28 (EV)	This Study
AH3052	MRSA Δ*spa*	(95)
NE611	*S. aureus* JE2 *tarM::*Tn	(77, 78)
NE942	*S. aureus* JE2 *tarS::*Tn	(77, 78)
AH6399	LAC *dltA::*Spec	This Study
AH6480	LAC *tarM::*Tn	This Study
AH6483	LAC *tarS::*Tn	This Study
TJE292	MRSA *sasF::*Tn/ pCM28::*sasF*	This Study
TJE357	MRSA *sasF::*Tn/ pEPSA5 (EV)	This Study
AH6823	LAC Δ*spa sbi::*Tet	This Study
TJE433	LAC Δ*spa sbi::*Tet/pEPSA5::*sasF*	This Study
TJE414	MRSA (WT)/pEPSA5::*sasF*	This Study
TJE445	MRSA Δ*spa sbi*::Tet *sasF*::Tn	This Study
AH2967	*S. aureus* RN4220/pTET	Ken Bayles
AH0203	*S. aureus* RN4220	Lab Stock
AH4807	LAC LL29::pHC125	(81)
TJE172	MRSA *sasF::*Tn LL29::pHC125	This Study
AH6491	*S. aureus* P68	Félix d'Hérelle Center

NE500	*S. aureus* JE2 *sasF::*Tn	(77, 78)
TJE141	AH1263/pCM29::P_{sasF}::GFP	This Study
<u>Plasmids</u><br>pCM28::*sasF*	Complementation vector	This study
pEPSA5 (EV)	Empty overexpression vector	(96)
pCM28 (EV)	Empty complementation vector	(92)
pEPSA5::*sasF*	SasF overexpression vector	This study
pTET	Transposon marker swap plasmid	(97)
pTEV5 (EV)	Empty protein expression vector	(79)
pTEV5::*sasF*$^{\Delta N \Delta C}$	SasF expression vector. N-terminal his tag.	This study
pHC125	Constitutive bioluminescence reporter vector. Chromosomally integrated.	(81)
pCM29::P_{sasF}	GFP reporter plasmid. GFP driven off P_{sasF}.	This study
pCM29	GFP reporter plasmid with GFP driven off of P_{sarA1}	(91)

Table S2: List Oligonucleotide Primers Used

Primer Name	Sequence (5'-3')	Source
sasF_Tn_F	TCCTTTACTTGTGTAGTTCTAAG	This Study
sasF_Tn_R	TAAGTGAGGAAGGAGTATGTTG	This Study
pTEV5_SasF_deltaNterminus _F	TAAACACGGCTAGCATTGAAATGAACGGTCAAAGTGATATAG	This Study
pTEV5_SasF_deltaCterminus_R_BamHI	AGCGGGATGGATCCTTATTATTATCGATCTAGTAAACTTGGACGATTTAC	This Study
pCM28MCS_seq_F	GGCTCATATTTGCTTTTTAAAAGC	This Study
pCM28_seq_F	AAGTTATTGTGATGACGACGA	This Study
pCM28_seq_R	CAAACCGCCTCTCCCCGC	This Study
pCM28MCS_seq_R2	GGCTCGTATGTTGTGTGGAATTG	This Study
tarM_Tn_F	AAAATTAAATGGATATTTTATTGCGTCAATTATAAGCCC	This Study
tarM_Tn_R	GTGGTTATGAATTTGAAATCATACATATCG	This Study
dltA_SpecDelet_F	TAAAGGCGTTTTTACTAAGAACTTTAAG	This Study
dltA_SpecDelet_R	AGGAATACTGAATTATTTATAATCAGTTATTACC	This Study
tarS_Tn_F	GATAAACATTTTAAATATCAAGATGGC	This Study
tarS_Tn_R	TTATTTATCATTATAGATAACCTCCTC	This Study
SasFComp_F	CAGAAGCTTGCATGCCTGCACACTTTATTACTCGAGGAGACATC	This Study
SasFComp_R	TTCATGATCGAATGCTAGCGAATGCGGTGGAACAAGCG	This Study
pEPSA5_F	ATTACAGCAGTCTGAGTTATAAAATAG	This Study
pEPSA5_R	AATTCTGTTTTATCAGACCGC	This Study
sbi_Tn_F	AAATTAAATGAGATTTCTACTCTACCAAACTCTCTTCG	This Study
sbi_Tn_R	GTGCTTGTAGTGGAAAAGGAATGG	This Study
SasF_SacI_F	TAAACACGGAGCTCATGTAATTCTTTGAAGGAAGTAAGTGAGG	This Study
SasF_BamHI_R	AGCGGGATGGATCCCTCCTTTACTTGTGTAGTTCTAAG	This Study
PsasF_XbaI_F	ATCTCCGGTCTAGAAAGGAAAAAGCAGTCTAAAATAAAAC	This Study
PsasF_KpnI_R	TTCATGCTGGTACCAAAGAATTACATACTATTATATACCTGTTTAC	This Study

REFERENCES

1. G. J. Moran, A. Krishnadasan, R. J. Gorwitz, G. E. Fosheim, L. K. McDougal, R. B. Carey, D. A. Talan, EMERGEncy ID Net Study Group, Methicillin-resistant *S. aureus* infections among patients in the emergency department. *N. Engl. J. Med.* **355**, 666–674 (2006).

2. J. Kluytmans, A. van Belkum, H. Verbrugh, Nasal carriage of *Staphylococcus aureus*: Epidemiology, underlying mechanisms, and associated risks. *Clin. Microbiol. Rev.* **10**, 505–520 (1997).

3. P. Ogonowska, Y. Gilaberte, W. Barańska-Rybak, J. Nakonieczna, Colonization with *Staphylococcus aureus* in atopic dermatitis patients: Attempts to reveal the unknown. *Front. Microbiol.* **11**, 567090 (2020).

4. P. H. Hoeger, W. Lenz, A. Boutonnier, J. M. Fournier, Staphylococcal skin colonization in children with atopic dermatitis: Prevalence, persistence, and transmission of toxigenic and nontoxigenic strains. *J. Infect. Dis.* **165**, 1064–1068 (1992).

5. C. von Eiff, K. Becker, K. Machka, H. Stammer, G. Peters, Nasal carriage as a source of *Staphylococcus aureus* bacteremia. Study Group. *N. Engl. J. Med.* **344**, 11–16 (2001).

6. J. P. O'Gara, Into the storm: Chasing the opportunistic pathogen *Staphylococcus aureus* from skin colonisation to life-threatening infections. *Environ. Microbiol.* **19**, 3823–3833 (2017).

7. T. J. Enroth, M. M. Severn, F. G. Costa, A. R. Bovee, R. V. Wilkening, D. T. Nguyen, C. Langouët-Astrié, A. R. Horswill, Global changes in *Staphylococcus aureus* virulence and metabolism during colonization of healthy skin. *Infect. Immun.* **93**, e00028–e00025 (2025).

8. A. M. Ibrahim, D. Missiakas, A novel polysaccharide in the envelope of *S. aureus* influences the septal secretion of preproteins with a YSIRK/GXXS motif. *J. Bacteriol.* **207**, e0047824 (2025).

9. A. DeDent, T. Bae, D. M. Missiakas, O. Schneewind, Signal peptides direct surface proteins to two distinct envelope locations of *Staphylococcus aureus*. *EMBO J.* **27**, 2656–2668 (2008).

10. O. Schneewind, D. Missiakas, Sortases, surface proteins, and their roles in *Staphylococcus aureus* disease and vaccine development. *Microbiol. Spectr.* **7**, 10.1128/microbiolspec.psib-0004-2018 (2019).

11. A. R. Cruz, J. A. G. van Strijp, F. Bagnoli, A. G. O. Manetti, Virulence gene expression of *Staphylococcus aureus* in human skin. *Front. Microbiol.* **12**, 692023 (2021).

12. M. G. Lei, R. K. Gupta, C. Y. Lee, Proteomics of *Staphylococcus aureus* biofilm matrix in a rat model of orthopedic implant-associated infection. *PLOS ONE* **12**, e0187981 (2017).

13. J. G. Kenny, D. Ward, E. Josefsson, I.-M. Jonsson, J. Hinds, H. H. Rees, J. A. Lindsay, A. Tarkowski, M. J. Horsburgh, The *Staphylococcus aureus* response to unsaturated long chain free fatty acids: Survival mechanisms and virulence implications. *PLOS ONE* **4**, e4344 (2009).

14. S. Brown, G. Xia, L. G. Luhachack, J. Campbell, T. C. Meredith, C. Chen, V. Winstel, C. Gekeler, J. E. Irazoqui, A. Peschel, S. Walker, Methicillin resistance in *Staphylococcus aureus* requires glycosylated wall teichoic acids. *Proc. Natl. Acad. Sci. U.S.A.* **109**, 18909–18914 (2012).

15. G. Xia, L. Maier, P. Sanchez-Carballo, M. Li, M. Otto, O. Holst, A. Peschel, Glycosylation of wall teichoic acid in *Staphylococcus aureus* by TarM. *J. Biol. Chem.* **285**, 13405–13415 (2010).

16. L. Mechler, E. J. Bonetti, S. Reichert, M. Flötenmeyer, J. Schrenzel, R. Bertram, P. François, F. Götz, Daptomycin tolerance in the *Staphylococcus aureus* pitA6 mutant is due to upregulation of the *dlt* operon. *Antimicrob. Agents Chemother.* **60**, 2684–2691 (2016).

17. A. Peschel, M. Otto, R. W. Jack, H. Kalbacher, G. Jung, F. Götz, Inactivation of the dlt operon in *Staphylococcus aureus* confers sensitivity to defensins, protegrins, and other antimicrobial peptides. *J. Biol. Chem.* **274**, 8405–8410 (1999).

18. A. Peschel, C. Vuong, M. Otto, F. Götz, The D-alanine residues of *Staphylococcus aureus* teichoic acids alter the susceptibility to vancomycin and the activity of autolytic enzymes. *Antimicrob. Agents Chemother.* **44**, 2845–2847 (2000).

19. T. Kohler, C. Weidenmaier, A. Peschel, Wall teichoic acid protects *Staphylococcus aureus* against antimicrobial fatty acids from human skin. *J. Bacteriol.* **191**, 4482–4484 (2009).

20. A. E. Paharik, A. R. Horswill, The staphylococcal biofilm: Adhesins, regulation, and host response. *Microbiol. Spect.* **4**, 10.1128/9781555819286.ch19 (2016).

21. L. J. L. Pizauro, C. C. De Almeida, S. R. Silva, J. I. Macinnes, A. M. Kropinski, L. F. Zafalon, F. A. De Avila, A. De Mello Varani, Genomic comparisons and phylogenetic analysis of mastitis-related staphylococci with a focus on adhesion, biofilm, and related regulatory genes. *Sci. Rep.* **11**, 17392 (2021).

22. R. A. Rodrigues, L. J. L. Pizauro, A. M. Varani, C. C. de Almeida, S. R. Silva, M. V. Cardozo, J. I. MacInnes, A. M. Kropinski, P. C. Melo, F. A. Avila, Comparative genomics study of *Staphylococcus aureus* isolated from cattle and humans reveals virulence patterns exclusively associated with bovine clinical mastitis strains. *Front. Microbiol.* **13**, 1033675 (2022).

23. L. M. Schlecht, B. M. Peters, B. P. Krom, J. A. Freiberg, G. M. Hänsch, S. G. Filler, M. A. Jabra-Rizk, M. E. Shirtliff, Systemic *Staphylococcus aureus* infection mediated by *Candida albicans* hyphal invasion of mucosal tissue. *Microbiology* **161**, 168–181 (2015).

24. T. J. Foster, J. A. Geoghegan, V. K. Ganesh, M. Höök, Adhesion, invasion and evasion: The many functions of the surface proteins of *Staphylococcus aureus*. *Nat. Rev. Microbiol.* **12**, 49–62 (2014).

25. J. Abramson, J. Adler, J. Dunger, R. Evans, T. Green, A. Pritzel, O. Ronneberger, L. Willmore, A. J. Ballard, J. Bambrick, S. W. Bodenstein, D. A. Evans, C.-C. Hung, M. O'Neill, D. Reiman, K. Tunyasuvunakool, Z. Wu, A. Žemgulytė, E. Arvaniti, C. Beattie, O. Bertolli, A. Bridgland, A. Cherepanov, M. Congreve, A. I. Cowen-Rivers, A. Cowie, M. Figurnov, F. B. Fuchs, H. Gladman, R. Jain, Y. A. Khan, C. M. R. Low, K. Perlin, A. Potapenko, P. Savy, S. Singh, A. Stecula, A. Thillaisundaram, C. Tong, S. Yakneen, E. D.

Zhong, M. Zielinski, A. Žídek, V. Bapst, P. Kohli, M. Jaderberg, D. Hassabis, J. M. Jumper, Accurate structure prediction of biomolecular interactions with AlphaFold 3. *Nature* **630**, 493–500 (2024).

26. K. B. Mills, J. J. Maciag, C. Wang, J. A. Crawford, T. J. Enroth, K. C. Keim, Y. F. Dufrêne, D. A. Robinson, P. D. Fey, A. B. Herr, A. R. Horswill, *Staphylococcus aureus* skin colonization is mediated by SasG lectin variation. *Cell Rep.* **43**, 114022 (2024).

27. S. Knox, N. M. O'Boyle, Skin lipids in health and disease: A review. *Chem. Phys. Lipids* **236**, 105055 (2021).

28. S. Ní Raghallaigh, K. Bender, N. Lacey, L. Brennan, F. C. Powell, The fatty acid profile of the skin surface lipid layer in papulopustular rosacea. *Br. J. Dermatol.* **166**, 279–287 (2012).

29. C. Weidenmaier, A. Peschel, Teichoic acids and related cell-wall glycopolymers in Gram-positive physiology and host interactions. *Nat. Rev. Microbiol.* **6**, 276–287 (2008).

30. R. van Dalen, A. Peschel, N. M. van Sorge, Wall teichoic acid in *Staphylococcus aureus* host interaction. *Trends Microbiol.* **28**, 985–998 (2020).

31. M. Gross, S. E. Cramton, F. Götz, A. Peschel, Key role of teichoic acid net charge in *Staphylococcus aureus* colonization of artificial surfaces. *Infect. Immun.* **69**, 3423–3426 (2001).

32. T. Suzuki, J. Campbell, Y. Kim, J. G. Swoboda, E. Mylonakis, S. Walker, M. S. Gilmore, Wall teichoic acid protects *Staphylococcus aureus* from inhibition by Congo red and other dyes. *J. Antimicrob. Chemother.* **67**, 2143–2151 (2012).

33. D. Kraus, S. Herbert, S. A. Kristian, A. Khosravi, V. Nizet, F. Götz, A. Peschel, The GraRS regulatory system controls *Staphylococcus aureus* susceptibility to antimicrobial host defenses. *BMC Microbiol.* **8**, 85 (2008).

34. I. C. Hancock, G. Wiseman, J. Baddiley, Biosynthesis of the unit that links teichoic acid to the bacterial wall: Inhibition by tunicamycin. *FEBS Lett.* **69**, 75–80 (1976).

35. J. Campbell, A. K. Singh, J. P. Santa Maria Jr., Y. Kim, S. Brown, J. G. Swoboda, E. Mylonakis, B. J. Wilkinson, S. Walker, Synthetic lethal compound combinations reveal a fundamental connection between wall teichoic acid and peptidoglycan biosyntheses in *Staphylococcus aureus*. *ACS Chem. Biol.* **6**, 106–116 (2011).

36. J. G. Swoboda, T. C. Meredith, J. Campbell, S. Brown, T. Suzuki, T. Bollenbach, A. J. Malhowski, R. Kishony, M. S. Gilmore, S. Walker, Discovery of a small molecule that blocks wall teichoic acid biosynthesis in *Staphylococcus aureus*. *ACS Chem. Biol.* **4**, 875–883 (2009).

37. Y. Lu, F. Chen, Q. Zhao, Q. Cao, R. Chen, H. Pan, Y. Wang, H. Huang, R. Huang, Q. Liu, M. Li, T. Bae, H. Liang, L. Lan, Modulation of MRSA virulence gene expression by the wall teichoic acid enzyme TarO. *Nat. Commun.* **14**, 1594 (2023).

38. S. H. Lee, H. Wang, M. Labroli, S. Koseoglu, P. Zuck, T. Mayhood, C. Gill, P. Mann, X. Sher, S. Ha, S.-W. Yang, M. Mandal, C. Yang, L. Liang, Z. Tan, P. Tawa, Y. Hou, R. Kuvelkar, K. DeVito, X. Wen, J. Xiao, M. Batchlett, C. J. Balibar, J. Liu, J. Xiao, N. Murgolo, C. G. Garlisi, P. R. Sheth, A. Flattery, J. Su, C. Tan, T. Roemer, TarO-specific inhibitors of wall teichoic acid biosynthesis restore β-lactam efficacy against methicillin-resistant staphylococci. *Sci. Transl. Med.* **8**, 329ra32 (2016).

39. N. T. Reichmann, C. P. Cassona, A. Gründling, Revised mechanism of D-alanine incorporation into cell wall polymers in Gram-positive bacteria. *Microbiology* **159**, 1868–1877 (2013).

40. M. Kovács, A. Halfmann, I. Fedtke, M. Heintz, A. Peschel, W. Vollmer, R. Hakenbeck, R. Brückner, A functional *dlt* operon, encoding proteins required for incorporation of D-alanine in teichoic acids in Gram-positive bacteria, confers resistance to cationic antimicrobial peptides in *Streptococcus pneumoniae*. *J. Bacteriol.* **188**, 5797–5805 (2006).

41. R. L. Gallo, K. J. Kim, M. Bernfield, C. A. Kozak, M. Zanetti, L. Merluzzi, R. Gennaro, Identification of CRAMP, a cathelin-related antimicrobial peptide expressed in the embryonic and adult mouse. *J. Biol. Chem.* **272**, 13088–13093 (1997).

42. H. Ingmer, D. Gerlach, C. Wolz, Temperate phages of *Staphylococcus aureus*. *Microbiol. Spectr.* **7**, 10.1128/microbiolspec.gpp1123-0058-2018 (2019).

43. S. Dong, O. N. Chesnokova, C. L. Turnbough Jr., D. G. Pritchard, Identification of the UDP-*N*-acetylglucosamine 4-epimerase involved in exosporium protein glycosylation in *Bacillus anthracis*. *J. Bacteriol.* **191**, 7094–7101 (2009).

44. Y.-W. Nam, M. Nishimoto, T. Arakawa, M. Kitaoka, S. Fushinobu, Structural basis for broad substrate specificity of UDP-glucose 4-epimerase in the human milk oligosaccharide catabolic pathway of *Bifidobacterium longum*. *Sci. Rep.* **9**, 11081 (2019).

45. H. Guo, L. Li, P. G. Wang, Biochemical characterization of UDP-GlcNAc/Glc 4-epimerase from *Escherichia coli* O86:B7. *Biochemistry* **45**, 13760–13768 (2006).

46. S. T. Allard, M. F. Giraud, J. H. Naismith, Epimerases: Structure, function and mechanism. *Cell. Mol. Life Sci.* **58**, 1650–1665 (2001).

47. K. E. Medvedev, L. N. Kinch, R. Dustin Schaeffer, J. Pei, N. V. Grishin, A fifth of the protein world: Rossmann-like proteins as an evolutionarily successful structural unit. *J. Mol. Biol.* **433**, 166788 (2021).

48. M. Blum, A. Andreeva, L. C. Florentino, S. R. Chuguransky, T. Grego, E. Hobbs, B. L. Pinto, A. Orr, T. Paysan-Lafosse, I. Ponamareva, G. A. Salazar, N. Bordin, P. Bork, A. Bridge, L. Colwell, J. Gough, D. H. Haft, I. Letunic, F. Llinares-López, A. Marchler-Bauer, L. Meng-Papaxanthos, H. Mi, D. A. Natale, C. A. Orengo, A. P. Pandurangan, D. Piovesan, C. Rivoire, J. A. Christian, N. Sigrist, F. T.-N. Thanki, P. D. Thomas, S. C. E. Tosatto, C. H. Wu, A. Bateman, InterPro: The protein sequence classification resource in 2025. *Nucleic Acids Res.* **53**, D444–D456 (2025).

49. T. Paysan-Lafosse, A. Andreeva, M. Blum, S. R. Chuguransky, T. Grego, B. L. Pinto, G. A. Salazar, M. L. Bileschi, F. Llinares-López, L. Meng-Papaxanthos, L. J. Colwell, N. V. Grishin, R. D. Schaeffer, D. Clementel, C. E. Silvio, E. Tosatto, V. Sonnhammer, A. B. Wood, The Pfam protein families database: Embracing AI/ML. *Nucleic Acids Res.* **53**, D523–D534 (2025).

50. A. Irimia, D. Madern, G. Zaccaï, F. M. Vellieux, Methanoarchaeal sulfolactate dehydrogenase: Prototype of a new family of NADH-dependent enzymes. *EMBO J.* **23**, 1234–1244 (2004).

51. A. H. Westphal, D. Tischler, F. Heinke, S. Hofmann, J. A. D. Gröning, D. Labudde, W. J. H. van Berkel, Pyridine nucleotide coenzyme specificity of *p*-hydroxybenzoate hydroxylase and related flavoprotein monooxygenases. *Front. Microbiol.* **9**, 3050 (2018).

52. H. Muramatsu, H. Mihara, M. Goto, I. Miyahara, K. Hirotsu, T. Kurihara, N. Esaki, A new family of NAD(P)H-dependent oxidoreductases distinct from conventional Rossmann-fold proteins. *J. Biosci. Bioeng.* **99**, 541–547 (2005).

53. F. Scheuplein, N. Schwarz, S. Adriouch, C. Krebs, P. Bannas, B. Rissiek, M. Seman, F. Haag, F. Koch-Nolte, NAD^+ and ATP released from injured cells induce $P2X_7$-dependent shedding of CD62L and externalization of phosphatidylserine by murine T cells. *J. Immunol.* **182**, 2898–2908 (2009).

54. S. Adriouch, S. Hubert, S. Pechberty, F. Koch-Nolte, F. Haag, M. Seman, NAD^+ released during inflammation participates in T cell homeostasis by inducing ART2-mediated death of naive T cells in vivo. *J. Immunol.* **179**, 186–194 (2007).

55. V. Audrito, V. G. Messana, L. Brandimarte, S. Deaglio, The extracellular NADome modulates immune responses. *Front. Immunol.* **12**, 704779 (2021).

56. J. Valladeau, O. Ravel, C. Dezutter-Dambuyant, K. Moore, M. Kleijmeer, Y. Liu, V. Duvert-Frances, C. Vincent, D. Schmitt, J. Davoust, C. Caux, S. Lebecque, S. Saeland, Langerin, a novel C-type lectin specific to Langerhans cells, is an endocytic receptor that induces the formation of Birbeck granules. *Immunity* **12**, 71–81 (2000).

57. J. Kwiecinski, T. Jin, E. Josefsson, Surface proteins of *Staphylococcus aureus* play an important role in experimental skin infection. *APMIS* **122**, 1240–1250 (2014).

58. X. Zheng, G. Marsman, K. A. Lacey, J. R. Chapman, C. Goosmann, B. M. Ueberheide, V. J. Torres, The cell envelope of *Staphylococcus aureus* selectively controls the sorting of virulence factors. *Nat. Commun.* **12**, 6193 (2021).

59. P. Hanson, C. J. Philp, H. S. Randeva, S. James, J. P. O'Hare, T. Meersmann, G. E. Pavlovskaya, T. M. Barber, Sodium in the dermis colocates to glycosaminoglycan scaffold, with diminishment in type 2 diabetes mellitus. *JCI Insight* **6**, e145470 (2021).

60. L. B. Baker, P. J. D. De Chavez, R. P. Nuccio, S. D. Brown, M. A. King, B. C. Sopeña, K. A. Barnes, Explaining variation in sweat sodium concentration: Effect of individual characteristics and exercise, environmental, and dietary factors. *J. Appl. Physiol.* **133**, 1250–1259 (2022).

61. J. Matthias, J. Maul, R. Noster, H. Meinl, Y.-Y. Chao, H. Gerstenberg, F. Jeschke, G. Gasparoni, A. Welle, J. Walter, K. Nordström, K. Eberhardt, D. Renisch, S. Donakonda, P. Knolle, D. Soll, S. Grabbe, N. Garzorz-Stark, K. Eyerich, T. Biedermann, D. Baumjohann, C. E. Zielinski, Sodium chloride is an ionic checkpoint for human T_H2 cells and shapes the atopic skin microenvironment. *Sci. Transl. Med.* **11**, eaau0683 (2019).

62. K. Denda-Nagai, S. Aida, K. Saba, K. Suzuki, S. Moriyama, S. Oo-Puthinan, M. Tsuiji, A. Morikawa, Y. Kumamoto, D. Sugiura, A. Kudo, Y. Akimoto, H. Kawakami, N. V. Bovin, T. Irimura, Distribution and function of macrophage galactose-type C-type lectin 2 (MGL2/CD301b): Efficient uptake and presentation of glycosylated antigens by dendritic cells. *J. Biol. Chem.* **285**, 19193–19204 (2010).

63. N. M. Van Sorge, N. M. C. Bleumink, S. J. Van Vliet, E. Saeland, W.-L. Van Der Pol, Y. Van Kooyk, J. P. M. Van Putten, N-glycosylated proteins and distinct lipooligosaccharide glycoforms of Campylobacter jejuni target the human C-type lectin receptor MGL. *Cell. Microbiol.* **11**, 1768–1781 (2009).

64. Y. Manome-Zenke, K. Denda-Nagai, R. Murakami, M. Noji, N. Tsuneda, K. B. Ishii-Schrade, N. Kanomata, S. Arai, T. Irimura, S. Ikeda, Possible involvement of antigen-presenting cells expressing the macrophage galactose-type C-type lectin in inflammatory skin diseases. *J. Invest. Dermatol.* **143**, 1834–1838.e10 (2023).

65. M. E. Mnich, R. van Dalen, D. Gerlach, A. Hendriks, G. Xia, A. Peschel, J. A. G. van Strijp, N. M. van Sorge, The C-type lectin receptor MGL senses *N*-acetylgalactosamine on the unique *Staphylococcus aureus* ST395 wall teichoic acid. *Cell. Microbiol.* **21**, e13072 (2019).

66. V. Winstel, P. Sanchez-Carballo, O. Holst, G. Xia, A. Peschel, Biosynthesis of the unique wall teichoic acid of *Staphylococcus aureus* lineage ST395. *MBio* **5**, e00869 (2014).

67. D. Gerlach, Y. Guo, C. De Castro, S.-H. Kim, K. Schlatterer, F.-F. Xu, C. Pereira, P. H. Seeberger, S. Ali, J. Codée, W. Sirisarn, B. Schulte, C. Wolz, J. Larsen, A. Molinaro, B. L. Lee, G. Xia, T. Stehle, A. Peschel, Methicillin-resistant *Staphylococcus aureus* alters cell wall glycosylation to evade immunity. *Nature* **563**, 705–709 (2018).

68. R. van Dalen, M. M. Molendijk, S. Ali, K. P. M. van Kessel, P. Aerts, J. A. G. van Strijp, C. J. C. de Haas, J. Codée, N. M. van Sorge, Do not discard *Staphylococcus aureus* WTA as a vaccine antigen. *Nature* **572**, E1–E2 (2019).

69. B. Bloom, R. Schelonka, T. Kueser, W. Walker, E. Jung, D. Kaufman, K. Kesler, D. Roberson, J. Patti, S. Hetherington, INH-A21 Phase II Study Team, Multicenter study to assess safety and efficacy of INH-A21, a donor-selected human staphylococcal immunoglobulin, for prevention of nosocomial infections in very low birth weight infants. *Pediatr. Infect. Dis. J.* **24**, 858–866 (2005).

70. M. DeJonge, D. Burchfield, B. Bloom, M. Duenas, W. Walker, M. Polak, E. Jung, D. Millard, R. Schelonka, F. Eyal, Clinical trial of safety and efficacy of IHN-A21 for the prevention of nosocomial staphylococcal bloodstream infection in premature infants. *J. Pediatr.* **151**, 260–265.e1 (2007).

71. V. G. Fowler, K. B. Allen, E. D. Moreira, M. Moustafa, F. Isgro, H. W. Boucher, G. R. Corey, Y. Carmeli, R. Betts, J. S. Hartzel, I. S. F. Chan, T. B. McNeely, N. A. Kartsonis, D. Guris, M. T. Onorato, S. S. Smugar, M. J. Di Nubile, A. S.-T. Meulen, Effect of an investigational vaccine for preventing *Staphylococcus aureus* infections after cardiothoracic surgery: A randomized trial. *JAMA* **309**, 1368–1378 (2013).

72. M. Inoue, T. Yonemura, J. Baber, Y. Shoji, M. Aizawa, D. Cooper, J. Eiden, W. C. Gruber, K. U. Jansen, A. S. Anderson, A. Gurtman, Safety, tolerability, and immunogenicity of a novel 4-antigen *Staphylococcus aureus* vaccine (SA4Ag) in healthy Japanese adults. *Hum. Vaccin. Immunother.* **14**, 2682–2691 (2018).

73. P. Joglekar, S. Conlan, S. Q. Lee-Lin, C. Deming, S. S. Kashaf, H. H. Kong, J. A. Segre, Integrated genomic and functional analyses of human skin-associated *Staphylococcus* reveal extensive inter- and intra-species diversity. *Proc. Natl. Acad. Sci. U.S.A.* **120**, e2310585120 (2023).

74. S. A. Lynch, K. J. Helbig, The complex diseases of *Staphylococcus pseudintermedius* in canines: Where to next? *Vet. Sci.* **8**, 11 (2021).

75. K. Worthing, S. Pang, D. J. Trott, S. Abraham, G. W. Coombs, D. Jordan, L. McIntyre, M. R. Davies, J. Norris, Characterisation of *Staphylococcus felis* isolated from cats using whole genome sequencing. *Vet. Microbiol.* **222**, 98–104 (2018).

76. S. Taponen, K. Supré, V. Piessens, E. Van Coillie, S. De Vliegher, J. M. K. Koort, *Staphylococcus agnetis* sp. nov., a coagulase-variable species from bovine subclinical and mild clinical mastitis. *Int. J. Syst. Evol. Microbiol.* **62**, 61–65 (2012).

77. T. Bae, E. M. Glass, O. Schneewind, D. Missiakas, Generating a collection of insertion mutations in the *Staphylococcus aureus* genome using bursa aurealis. *Methods Mol. Biol.* **416**, 103–116 (2008).

78. P. D. Fey, J. L. Endres, V. K. Yajjala, T. J. Widhelm, R. J. Boissy, J. L. Bose, K. W. Bayles, A genetic resource for rapid and comprehensive phenotype screening of nonessential *Staphylococcus aureus* genes. *MBio* **4**, e00537–e00512 (2013).

79. C. J. Rocco, K. L. Dennison, V. A. Klenchin, I. Rayment, J. C. Escalante-Semerena, Construction and use of new cloning vectors for the rapid isolation of recombinant proteins from *Escherichia coli*. *Plasmid* **59**, 231–237 (2008).

80. N. P. King, T. Sakinç, N. L. Ben Zakour, M. Totsika, B. Heras, P. Simerska, M. Shepherd, S. G. Gatermann, S. A. Beatson, M. A. Schembri, Characterisation of a cell wall-anchored protein of *Staphylococcus saprophyticus* associated with linoleic acid resistance. *BMC Microbiol.* **12**, 8 (2012).

81. R. J. Miller, H. A. Crosby, K. Schilcher, Y. Wang, R. V. Ortines, M. Mazhar, D. A. Dikeman, B. L. Pinsker, I. D. Brown, D. P. Joyce, J. Zhang, N. K. Archer, H. Liu, M. P. Alphonse, J. Czupryna, W. R. Anderson, N. M. Bernthal, L. Fortuno-Miranda, J. W. M. Bulte, K. P. Francis, A. R. Horswill, L. S. Miller, Development of a *Staphylococcus aureus* reporter strain with click beetle red luciferase for enhanced in vivo imaging of experimental bacteremia and mixed infections. *Sci. Rep.* **9**, 16663 (2019).

82. A. S. Bayer, L. I. Kupferwasser, M. H. Brown, R. A. Skurray, S. Grkovic, T. Jones, K. Mukhopadhay, M. R. Yeaman, Low-level resistance of *Staphylococcus aureus* to thrombin-induced platelet microbicidal protein 1 in vitro associated with qacA gene carriage is independent of multidrug efflux pump activity. *Antimicrob. Agents Chemother.* **50**, 2448–2454 (2006).

83. K. Kho, T. C. Meredith, Extraction and analysis of bacterial teichoic acids. *Bio Protoc.* **8**, e3078 (2018).

84. W. F. Vranken, W. Boucher, T. J. Stevens, R. H. Fogh, A. Pajon, M. Llinas, E. L. Ulrich, J. L. Markley, J. Ionides, E. D. Laue, The CCPN data model for NMR spectroscopy: Development of a software pipeline. *Proteins* **59**, 687–696 (2005).

85. F. Delaglio, S. Grzesiek, G. W. Vuister, G. Zhu, J. Pfeifer, A. Bax, NMRPipe: A multidimensional spectral processing system based on UNIX pipes. *J. Biomol. NMR* **6**, 277–293 (1995).

86. M. Imber, V. V. Loi, S. Reznikov, V. N. Fritsch, A. J. Pietrzyk-Brzezinska, J. Prehn, C. Hamilton, M. C. Wahl, A. K. Bronowska, H. Antelmann, The aldehyde dehydrogenase AldA contributes to the hypochlorite defense and is redox-controlled by protein S-bacillithiolation in *Staphylococcus aureus*. *Redox Biol.* **15**, 557–568 (2018).

87. A. Ben Chorin, G. Masrati, A. Kessel, A. Narunsky, J. Sprinzak, S. Lahav, H. Ashkenazy, N. Ben-Tal, ConSurf-DB: An accessible repository for the evolutionary conservation patterns of the majority of PDB proteins. *Protein Sci.* **29**, 258–267 (2020).

88. J. A. F. Sutton, O. T. Carnell, L. Lafage, J. Gray, J. Biboy, J. F. Gibson, E. J. G. Pollitt, S. C. Tazoll, W. Turnbull, N. H. Hajdamowicz, B. Salamaga, G. R. Pidwill, A. M. Condliffe, S. A. Renshaw, W. Vollmer, S. J. Foster, *Staphylococcus aureus* cell wall structure and dynamics during host-pathogen interaction. *PLOS Pathog.* **17**, e1009468 (2021).

89. F. Madeira, N. Madhusoodanan, J. Lee, A. Eusebi, A. Niewielska, A. R. N. Tivey, R. Lopez, S. Butcher, The EMBL-EBI Job Dispatcher sequence analysis tools framework in 2024. *Nucleic Acids Res.* **52**, W521–W525 (2024).

90. X. Robert, C. Guillon, P. Gouet, FoldScript: A web server for the efficient analysis of AI-generated 3D protein models. *Nucleic Acids Res.* **53**, W277–W282 (2025).

91. Y. Y. Pang, J. Schwartz, M. Thoendel, L. W. Ackermann, A. R. Horswill, W. M. Nauseef, agr-Dependent interactions of *Staphylococcus aureus* USA300 with human polymorphonuclear neutrophils. *J. Innate Immun.* **2**, 546–559 (2010).

92. B. R. Boles, M. Thoendel, A. J. Roth, A. R. Horswill, Identification of genes involved in polysaccharide-independent *Staphylococcus aureus* biofilm formation. *PLOS ONE* **5**, e10146 (2010).

93. M. A. Benson, S. Lilo, G. A. Wasserman, M. Thoendel, A. Smith, A. R. Horswill, J. Fraser, R. P. Novick, B. Shopsin, V. J. Torres, *Staphylococcus aureus* regulates the expression and production of the staphylococcal superantigen-like secreted proteins in a Rot-dependent manner. *Mol. Microbiol.* **81**, 659–675 (2011).

94. L. Deng, K. Schilcher, L. R. Burcham, J. M. Kwiecinski, P. M. Johnson, S. R. Head, D. E. Heinrichs, A. R. Horswill, K. S. Doran, Identification of key determinants of *Staphylococcus aureus* vaginal colonization. *mBio* **10**, e02321-19 (2019).

95. C. B. Ibberson, C. L. Jones, S. Singh, M. C. Wise, M. E. Hart, D. V. Zurawski, A. R. Horswill, *Staphylococcus aureus* hyaluronidase is a CodY-regulated virulence factor. *Infect. Immun.* **82**, 4253–4264 (2014).

96. R. A. Forsyth, R. J. Haselbeck, K. L. Ohlsen, R. T. Yamamoto, H. Xu, J. D. Trawick, D. Wall, L. Wang, V. Brown-Driver, J. M. Froelich, K. G. C, P. King, M. M. Carthy, C. Malone, B. Misiner, D. Robbins, Z. Tan, Z.-Y. Z. Zy, G. Carr, D. A. Mosca, C. Zamudio, J. G. Foulkes, J. W. Zyskind, A genome-wide strategy for the identification of essential genes in *Staphylococcus aureus*. *Mol. Microbiol.* **43**, 1387–1400 (2002).

97. J. L. Bose, P. D. Fey, K. W. Bayles, Genetic tools to enhance the study of gene function and regulation in *Staphylococcus aureus*. *Appl. Environ. Microbiol.* **79**, 2218–2224 (2013).
